# Modular Attachment of Nanoparticles on Microparticle Supports via Multifunctional Polymers

**DOI:** 10.1021/acs.chemmater.3c00555

**Published:** 2023-04-26

**Authors:** Maximilian R. Bailey, Tobias A. Gmür, Fabio Grillo, Lucio Isa

**Affiliations:** Laboratory for Soft Materials and Interfaces, Department of Materials, ETH Zürich, Zürich 8093, Switzerland

## Abstract

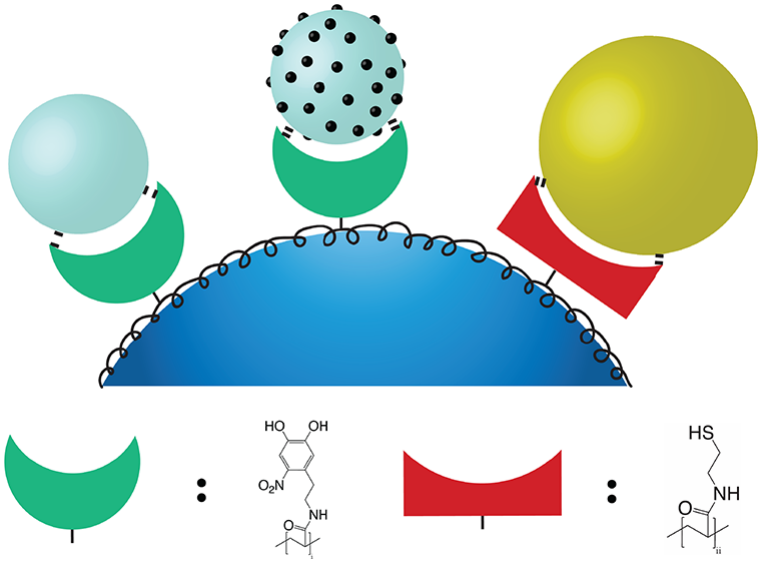

Nanoparticles are key to a range of applications, due to the properties that emerge as a result of their small size. However, their size also presents challenges to their processing and use, especially in relation to their immobilization on solid supports without losing their favorable functionalities. Here, we present a multifunctional polymer-bridge-based approach to attach a range of presynthesized nanoparticles onto microparticle supports. We demonstrate the attachment of mixtures of different types of metal-oxide nanoparticles, as well as metal-oxide nanoparticles modified with standard wet chemistry approaches. We then show that our method can also create composite films of metal and metal-oxide nanoparticles by exploiting different chemistries simultaneously. We finally apply our approach to the synthesis of designer microswimmers with decoupled mechanisms of steering (magnetic) and propulsion (light) via asymmetric nanoparticle binding, aka Toposelective Nanoparticle Attachment. We envision that this ability to freely mix available nanoparticles to produce composite films will help bridge the fields of catalysis, nanochemistry, and active matter toward new materials and applications.

## Introduction

The past decades have seen a rapid growth of research on the potential applications of nanoparticles^1^ and their assembly as “artificial atoms” into increasingly complex structures and composite materials.^2,3^ Motivated by favorable properties, such as the high density of active unsaturated atoms residing on their surface^4^ and discontinuous effects arising from their confined electronic band structure,^5^ nanoparticles and nanoparticle composites are of specific interest to fields ranging from catalysis^6^ to nanophotonics.^7^

One of the more promising aspects of using nanostructured materials is the ability to combine mixtures of different nanoparticles to improve the performances of the starting materials—or even observe emergent properties.^8,9^ This feature has been regularly exploited in heterogeneous photocatalysis, where the relatively poor visible light absorption and quantum efficiency of base photocatalysts such as TiO_2_ can be improved by incorporating cocatalyst metal nanoparticles.^10,11^ Typically, materials are functionalized with nanoparticles either by synthesizing the nanoparticles directly onto the support^12^ or by heteroaggregating presynthesized metal nanoparticles.^13^ In this second approach, the size and the geometry of the nanoparticles can be predefined, in turn providing greater control over their functionality^14−16^

However, there are significant hurdles still facing the application of nanoparticles to many real-world situations. One of the key issues is that their small size not only makes them hard to process, but also poses health and safety concerns. Furthermore, the recovery of nanosized objects from liquids is difficult, which introduces additional problems when applications require that the nanoparticles are suspended in a fluid.^17^ Nanoparticles are also more prone to aggregation due to their high surface energy,^1^ and thus more challenging to disperse in liquids than their larger counterparts.^18,19^ To overcome these and other challenges, nanoparticles are often supported on a secondary material.^20,21^

Among the various strategies for nanoparticle support, attaching them onto microparticles has received comparatively less attention. Nevertheless, microparticles possess significant favorable attributes as a support material. First and foremost, they enable dispersion in liquid media while retaining the high mass-transfer rates and high surface areas of the nanoparticles, with the advantage of their easy removal from suspension.^22^ For example, Marques et al. demonstrated that the advantages of slurry-type photocatalytic reactors could be retained without the need for a time-consuming and complex nanofiltration step to recover the catalyst upon attaching it onto microparticles.^23^ Moreover, the size, morphology, porosity, and composition of microparticles can also be adjusted by drawing upon the extensive and well-established literature of colloidal synthesis, allowing targeted development of the support material toward the desired application.

Strong attachment between functional nanoparticles and support materials is often achieved via thermal annealing. However, this can lead to sintering of the metal nanoparticles and the loss of desirable morphologies, reducing their overall performance.^15,24^ Polymers have been demonstrated as effective anchors without the need for further processing steps that are potentially detrimental to the nanoparticle properties.^25−27^ By avoiding harsh thermal or chemical treatments, polymers also enable the synthesis of “Janus” particles via the Pickering wax emulsion technique.^28−30^ The “patchiness” of such particles can lead to new properties interesting for a range of applications.^31^ For example, Synytska and co-workers demonstrated the benefits of using “hairy”, submicron catalytic Janus particles in interfacial catalysis, exploiting an asymmetry in the particle wetting properties and the distribution of metal nanoparticles grown in situ from solution, to better stabilize and catalyze reactions in emulsions.^32,33^ The asymmetric modification of solid microparticle supports with presynthesized functional nanoparticles is also highly advantageous for the synthesis of active matter systems.^34−38^ For example, the anisotropic distribution of catalytic material on a Janus microswimmer generates local gradients around its surface by decomposing “fuel”, resulting in self-phoretic motion.^39−42^ Currently, the synthesis of active Janus colloids suffers from several limitations, in particular, an over-reliance on physical vapor deposition methods for particle functionalization.^37^ Not only does this limit the scalability of Janus microswimmer synthesis, but also it significantly constrains the selection and quality of the materials and leads to poor reproducibility between batches.^43^ In contrast, by making use of presynthesized functional nanoparticles, favorable properties such as high crystallinity or specific material phases can be programmed into the microswimmer design, in-turn imparting improved catalytic efficiency or visible light activation to photoresponsive Janus colloids. This is particularly promising for the “chemistry-on-the-fly” concept,^44^ as the nanocatalysts can be targeted toward fuels generating useful chemical products as a byproduct of microswimmer propulsion. Thus, nanoparticle-functionalized microswimmers could realize their potential as catalyst-stirrers, locally mixing solutions while acting as motile catalysts, thereby enhancing the overall reaction efficiency.^45^

Here, we propose a flexible platform to achieve the versatile and modular attachment of a diverse range of nanoparticles onto SiO_2_ microparticles. To this end, we exploit a multipurpose polymer bridge to attach mixtures of metal-oxide and metal nanoparticles onto microparticle supports. In brief, we postmodify poly(pentafluorophenyl acrylate) (pPFPAc) to obtain a polymer backbone with different chemical functional groups^46^ tailored toward the presynthesized nanoparticles of interest. We first demonstrate the versatility of our approach by attaching a mixture of metal-oxide nanoparticles, including magnetic iron oxide. We then show the generalizability of our method by attaching premodified TiO_2_ nanoparticles, drawing upon the extensive library of synthetic strategies in the literature to functionalize TiO_2_ P-25 with metal nanoparticles to obtain superior photocatalysts. We then highlight the modularity of our polymer bridge by including groups that enable nitro-catechol chelation and metal–thiolate chemistry to drive the cobinding of TiO_2_ P-25 aggregates and single Au nanoparticles onto SiO_2_ microparticles via simple bulk stirring. Finally, we demonstrate an application of our modular approach to nanoparticle attachment by extending our previously described Toposelective Nanoparticle Attachment (TNA^30^) to incorporate magnetic functionality into our microswimmers and thus obtain decoupled mechanisms of propulsion and steering. The intricate, 3D motion of the resultant multifunctional microswimmers is characterized using a Machine Learning algorithm that we previously trained on experimental data.^47^

## Results and Discussion

**Scheme 1:**
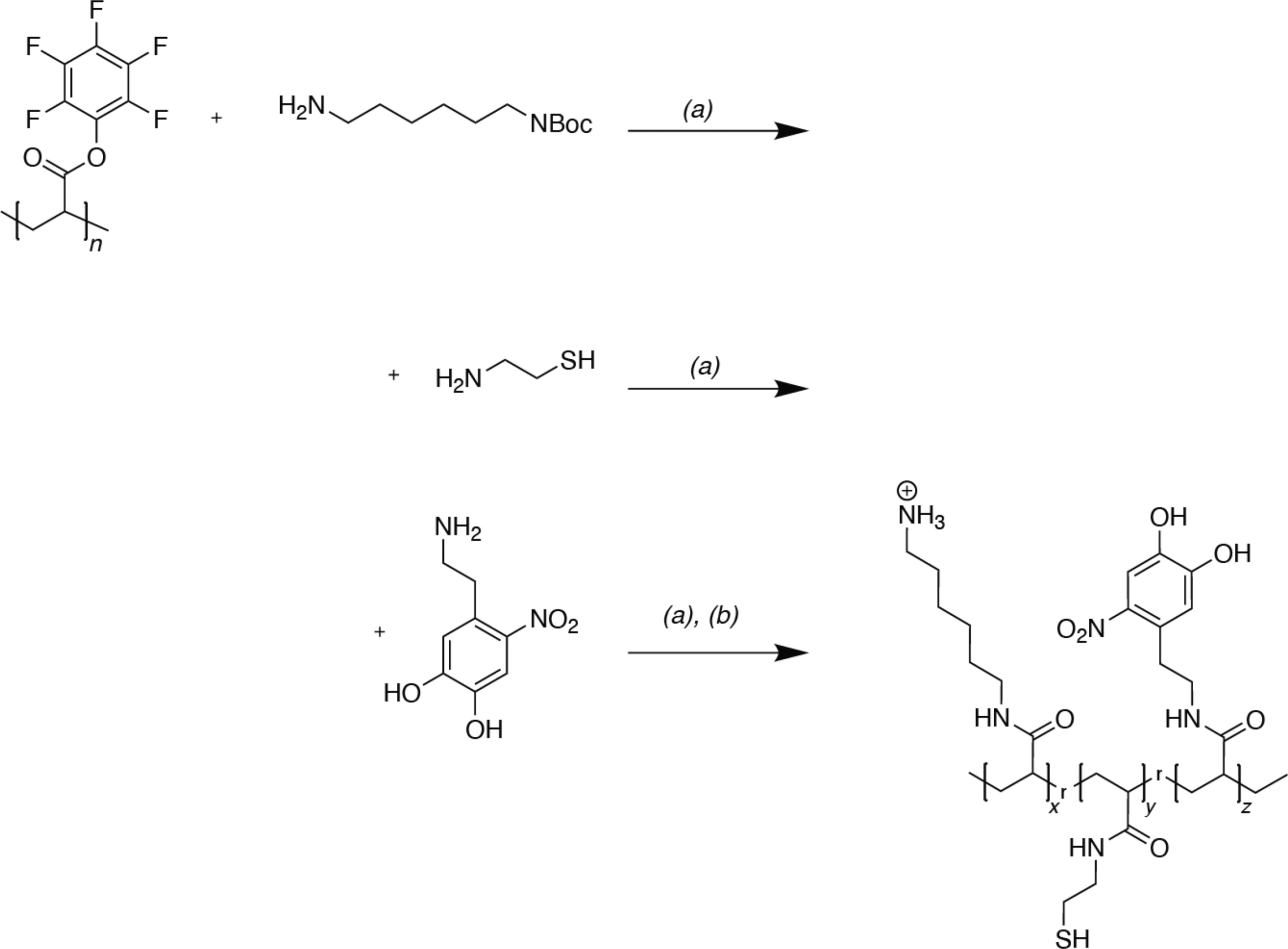
Synthesis Scheme for the Preparation of the Fully Modified Poly-(hexyl-6-amine, Ethylthiol, Nitrodopamine)-Acrylamide (a) DMF and TEA, 50° C; (b) DCM and TFA overnight, dialysis in H_2_O.

As mentioned in the Introduction, we use a postmodified poly(pentafluorophenyl acrylate) (pPFPAc) backbone as the core element of our functionalization strategy. The choice of a pFPAC backbone is specifically motivated by the presence of reactive ester linkages, which can be exchanged with amine-containing functional groups by nucleophilic substitution.^46^ Our approach thus hinges on this ability to postmodify the “Swiss army knife” PPFPAC backbone with various chemical functional groups, as detailed in Scheme 1, to firmly bind nanoparticles onto the microparticle supports in a one-pot bulk mixing step. As we show later, the chemical groups can be targeted toward specific metal surface chemistries of interest, providing a platform to obtain hybrid materials composed of different nanoparticles according to the desired application.^3^

In each version of the polymer, an electrostatic component is introduced via the presence of primary amine groups, which remain positively charged over a wide range of pH. Not only does this improve the polymer’s solubility in water, but also it assists the proper conformation and attachment of the polymer to negatively charged substrates.^26,46^ In this manner, the polymer is well-attached to the SiO_2_ particles. However, the firm attachment of the nanoparticles necessitates additional strongly binding functional groups to ensure that they are not easily removed from the SiO_2_ support. The ability of catechol groups to form strong bonds with metal-oxide surfaces is well characterized,^48^ and we have previously demonstrated the ability of nitro-catechol chelation chemistry to bind various phases of TiO_2_, as well as Fe_2_O_3_ and SrTiO_3_, onto SiO_2_ microparticles.^30^

### Co-attachment of Different Primary Metal-Oxide Nanoparticles

**Figure 1:**
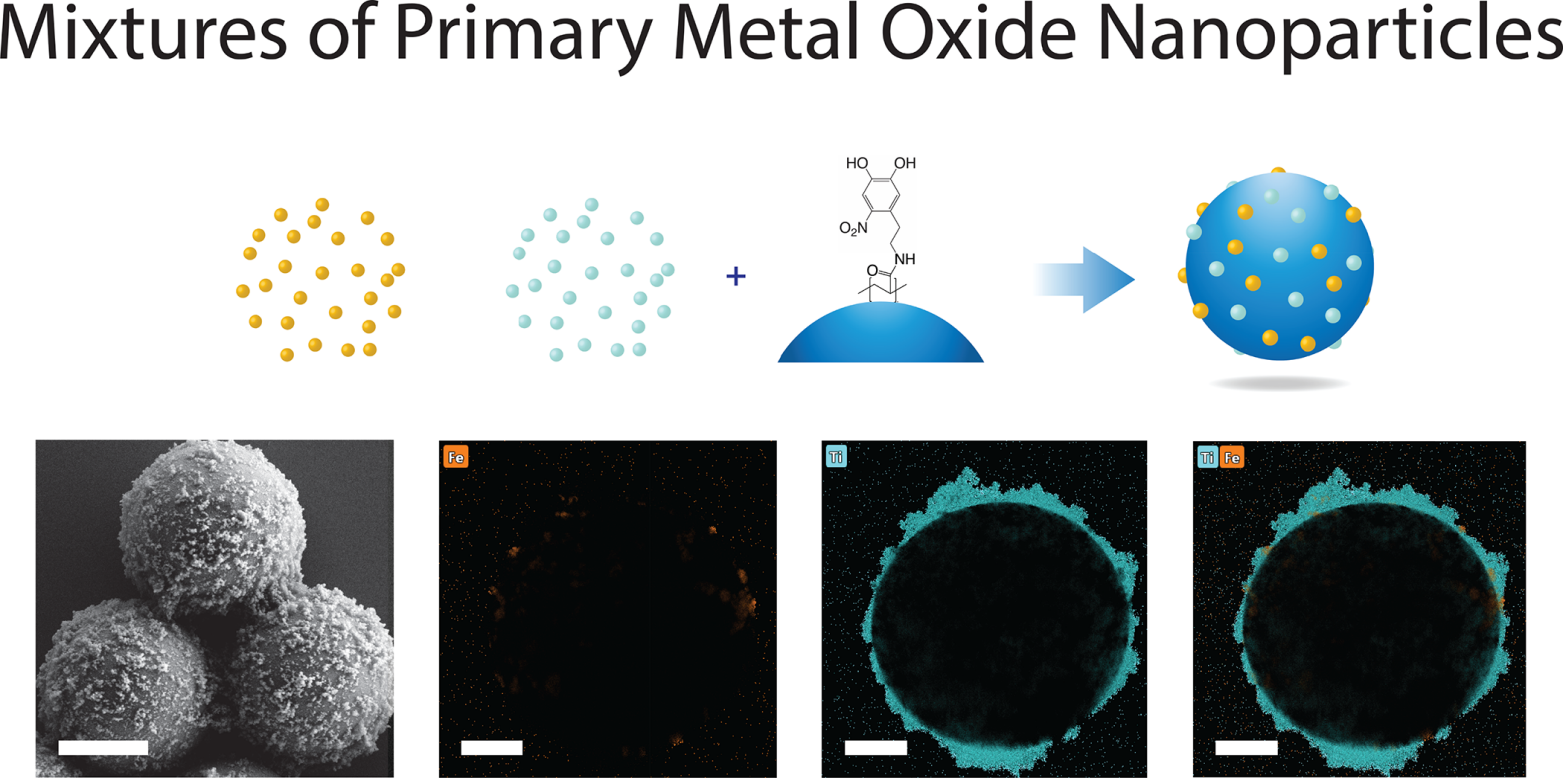
Top row: Attaching different metal-oxide nanoparticles in a single step via the nitrodopamine group of the polymer bridge. Bottom row: Attachment of a 9:1 mixture of TiO_2_ and Fe_2_O_3_ nanoparticles. The presence of both elements is visualized with EDX mapping. Left to right: HR-SEM (scale bar, 1000 nm) and STEM-EDX mapping of Fe, Ti, and Fe + Ti overlaid, respectively (scale bar, 500 nm).

Here, we first extend our previously described methodology to easily obtain microparticle supports with composite oxide nanoparticle thin films possessing multiple functionalities via an ex situ heteroaggreation process using the nitrodopamine group of our polymer bridge.^30^ We attach mixed films of TiO_2_ and Fe_2_O_3_ to SiO_2_ microparticles by simply adjusting the ratio of TiO_2_ and Fe_2_O_3_ nanoparticles initially added during the bulk heteroaggregation step.^3^ Incorporating magnetic functionality into micro- and nanoscale devices and catalysts is desirable due to the ease of their recovery after use.^27,49,50^ We thus incorporate a small quantity of Fe_2_O_3_ nanoparticles (9:1 TiO_2_:Fe_2_O_3_ added) and find both species present on the SiO_2_ support (see Figure 1, EDS mapping). We furthermore verify the presence of both metal oxides in the bulk sample by X-ray photoelectron spectroscopy (XPS) and demonstrate control over their relative surface coverage on the microparticle supports via the ratios of nanoparticles added (see Figures S1 and S2 and Table S1). Promisingly, the mild heteroaggregation process helps retain the magnetic and photocatalytic functionality of the supported nanoparticles (see Figures S3, S4, and S5).

### Attachment of Modified Metal-Oxide Nanoparticles

The performance of unfunctionalized TiO_2_ P-25 as a photocatalyst, with its large band gap (3.2 eV) and high rate of photogenerated charge recombination, has motivated a significant body of research aimed at optimizing its photocatalytic efficiency and visible light absorption.^10,11,17,51^ In particular, the use of metal, cocatalyst nanoparticles has been proposed to achieve these goals and make TiO_2_ a viable photocatalyst for applications ranging from H_2_ generation and CO_2_ reduction to pollution remediation.^51−53^ Therefore, it is important to verify that our polymer bridge-based approach to nanoparticle attachment is still viable after the surface modification of TiO_2_ from its “clean”, unfunctionalized commercial form using standard, wet-chemistry methods.

To this end, we premodify commercial TiO_2_ P-25 nanoparticles (Sigma-Aldrich, Aeroxide) with Pt and Au nanoparticles (M-TiO_2_) using established protocols from literature, prior to their attachment to the SiO_2_ microparticle supports via the polymer bridge. Specifically, we functionalize TiO_2_ P-25 with presynthesized Pt nanoparticles via solvent evaporation of the dispersed nanoparticles in MeOH, following the protocol found in ref (15) while Au nanoparticles are directly grown onto the TiO_2_ nanoparticles from solution under water reflux conditions, as outlined in ref (54). By using two different, but well-established, approaches to cocatalyst modification of a photocatalyst (solvent evaporation and deposition-precipitation, respectively), we aim to demonstrate the broad applicability of our approach to the support of functional photocatalysts.

**Figure 2:**
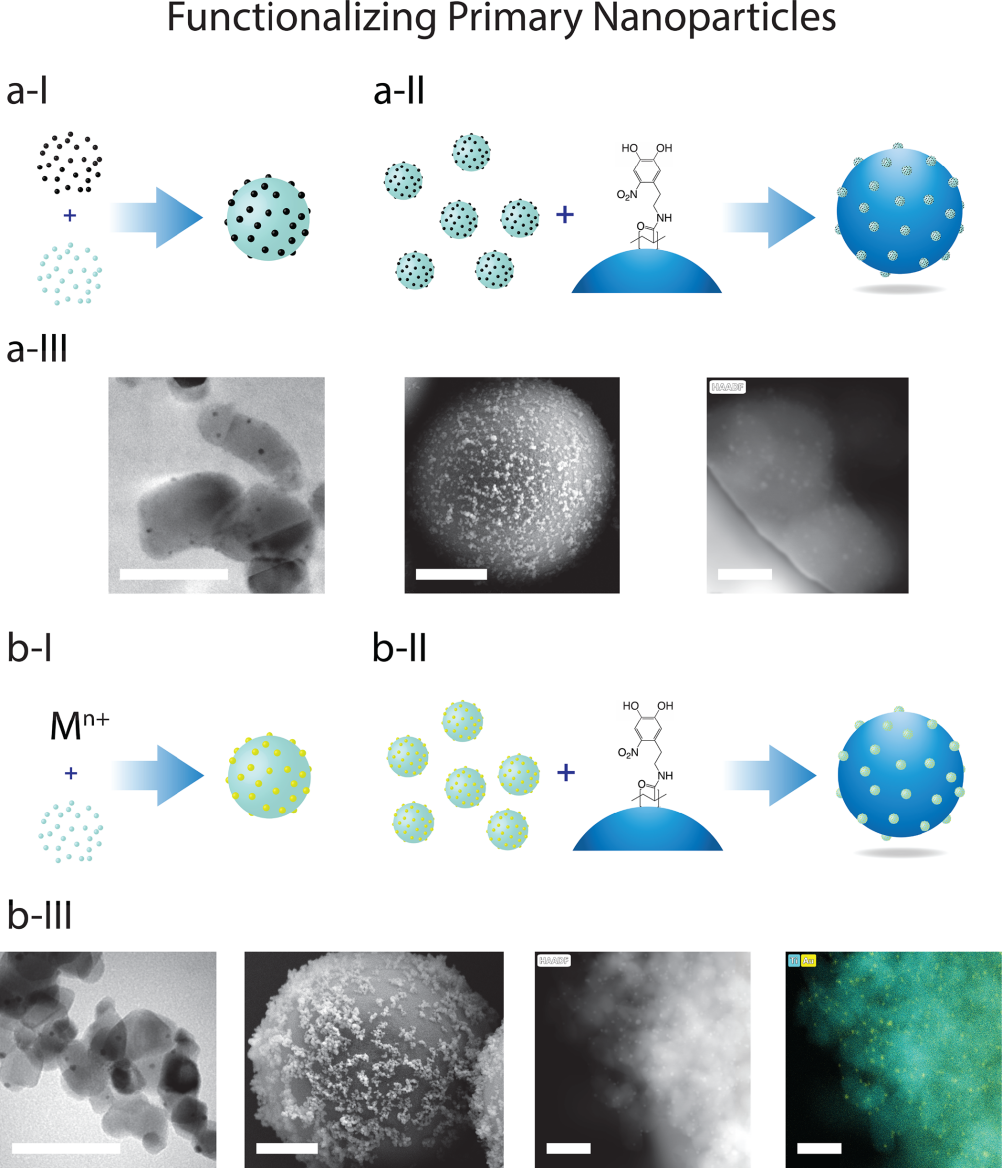
Modifying TiO_2_ before attachment onto the SiO_2_ microparticle supports. (a) Supporting TiO_2_–Pt on SiO_2_ microparticles. (a-I) TiO_2_ P-25 is modified with presynthesized Pt nanoparticles. (a-II) TiO_2_–Pt nanoparticles are supported on SiO_2_ microparticles via the nitrodopamine group on the polymer bridge. The HAADF-STEM image is processed to enhance contrast. (a-III) Left to right: HR-TEM (scale bar, 50 nm), HR-SEM (scale bar, 500 nm), and HAADF-STEM (scale bar, 15 nm). (b) Supporting TiO_2_–Au on SiO_2_ microparticles. (b-I) Au nanoparticles are grown on TiO_2_ P-25 in situ by deposition-precipitation. (b-II) TiO_2_–Au nanoparticles are supported on SiO_2_ microparticles via the nitrodopamine group on the polymer bridge. (b-III) Left to right: HR-TEM (scale bar, 50 nm), HR-SEM (scale bar, 500 nm), and HAADF-STEM and EDX mapping (scale bar, 50 nm).

Figure 2 presents both a schematic and experimental results for the two strategies of cocatalyst functionalization of TiO_2_ P-25 nanoparticles followed by their attachment to SiO_2_ microparticle supports. In Figure 2a, the functionalization of TiO_2_ nanoparticles with Pt nanoparticles via solvent evaporation from MeOH is presented in a-I, followed by their attachment under bulk stirring to SiO_2_ microparticle supports in a-II. The presence of Pt nanoparticles on the TiO_2_ P-25 is visually confirmed by HR-TEM Z-contrast imaging (see Figure 2a-III, left), while HAADF-STEM detects the presence of Pt nanoparticles (brighter spots) on the TiO_2_ aggregates attached to the SiO_2_ microparticle support (see Figure 2a-III, right). We note that STEM elemental mapping of the Pt nanoparticles is not possible; however, we observe L-edges corresponding to Pt (see Figure S6)

Likewise, the growth of Au nanoparticles from solution, following the recipe presented in ref (54), is demonstrated in Figure 2b. The presence of Au nanoparticles on the TiO_2_ P-25 is visually confirmed by HR-TEM Z-contrast imaging (see Figure 2b-III, left). We furthermore confirm that Au nanoparticles are present on the TiO_2_ aggregates attached to the SiO_2_ microparticle supports by HAADF-STEM and EDX mapping (see Figure 2b-III, middle right and right, and Figure S7 for further details). We thus demonstrate that the dispersion of TiO_2_ P-25 and Pt nanoparticles in MeOH, as well as the use of a urea precipitating agent for the controlled growth of Au nanoparticles onto the TiO_2_, does not affect their attachment via the nitro-catechol groups of the polymer-bridge onto the SiO_2_ microparticles. The presence of the metal cocatalysts on the TiO_2_ supported on the SiO_2_ microparticles, visualized by STEM imaging, confirms that we do not selectively bind unfunctionalized TiO_2_ onto the SiO_2_. In particular, we find the results presented in (a) promising, as the Co4Cat (colloids for catalysts) approach outlined by Quinson et al. is broadly applicable to a range of metallic and bimetallic nanoparticles,^15^ indicating the possibility of tailoring the supported nanoparticles toward a desired application.

### Co-attachment of Metallic and Metal-Oxide Nanoparticles

**Figure 3:**
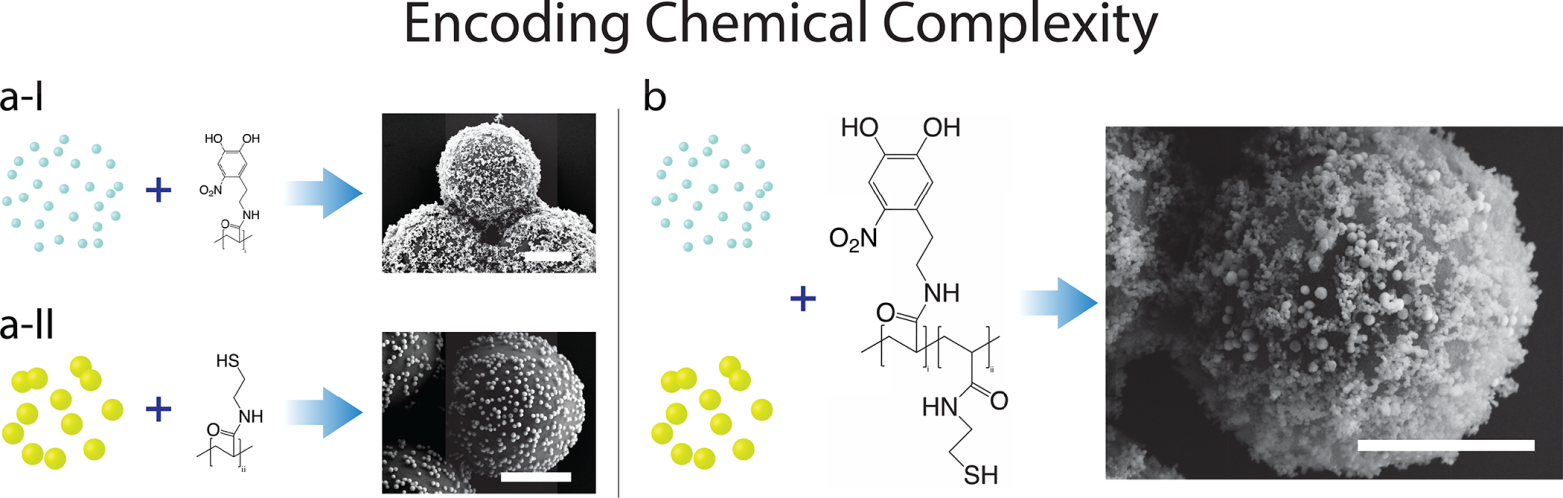
Incorporation of different nanoparticles via appropriate selection of chemical functional groups. (a-I) TiO_2_ P-25 nanoparticle aggregates and (a-II) Au single nanoparticles are attached to the SiO_2_ microparticle support via metal-oxide nitro-catechol chelation and metal–thiolate chemistry, respectively. (b) The combination of both chemical groups allows the attachment of the metal and metal-oxide nanoparticles using a single polymer bridge. The two nanoparticles can be clearly distinguished by their morphologies in SEM images. Scale bars denote 1000 nm.

Perhaps the most promising feature of our polymer-bridge mediated attachment of nanoparticles is the ability to encode mixtures of chemical functionalities into the “Swiss-army-knife” pPFPAC polymer backbone and, thus, obtain a truly modular approach to nanoparticle support via appropriate selection of the chemical groups. Specifically, we demonstrate this potential by incorporating thiol groups in addition to the nitro-catechol functionality to enable attachment of both metal-oxide and metal nanoparticles separately to the same SiO_2_ microparticle support. We choose thiol groups for their well-known ability to form strong bonds with a range of metal surfaces.^55^Figure 3 presents an overview of our proposed approach, first illustrating the separate attachment of nanoparticles (TiO_2_ and Au) via their respective, appropriately selected chemical groups (nitro-catechol and thiol), followed by the attachment of the combination via a polymer bridge containing both chemical functionalities. The morphological differences between the TiO_2_ P-25 nanoparticles (aggregates with primary nanoparticle size around 21 nm) and the Au nanoparticles (spherical, diameter ∼70 nm) allows their distinction by visual inspection. We furthermore verify the presence of both nanoparticle species in the bulk sample by XPS (see Figure S9 and Table S2). The relevant controls presented in Figure S8 demonstrate the importance of both the nitrodopamine and thiol groups for the attachment of metal-oxide and metal nanoparticles, respectively.

### Magnetically Responsive Photocatalytic Active Systems

Having demonstrated the possibilities for modular, nanoparticle attachment to obtain microparticle supports with multiple functionalities, we now demonstrate an application for this method, namely, by extending our previously described Toposelective Nanoparticle Attachment (TNA) method toward obtaining large quantities of Janus microswimmers.^30^ The simplicity of the above-described modular approach to nanoparticle attachment, which only requires heteroaggregation under bulk stirring, allows the easy transfer to the Pickering wax emulsion technique,^28,29^ to obtain Janus particles asymmetrically modified with presynthesized nanoparticles.

**Figure 4:**
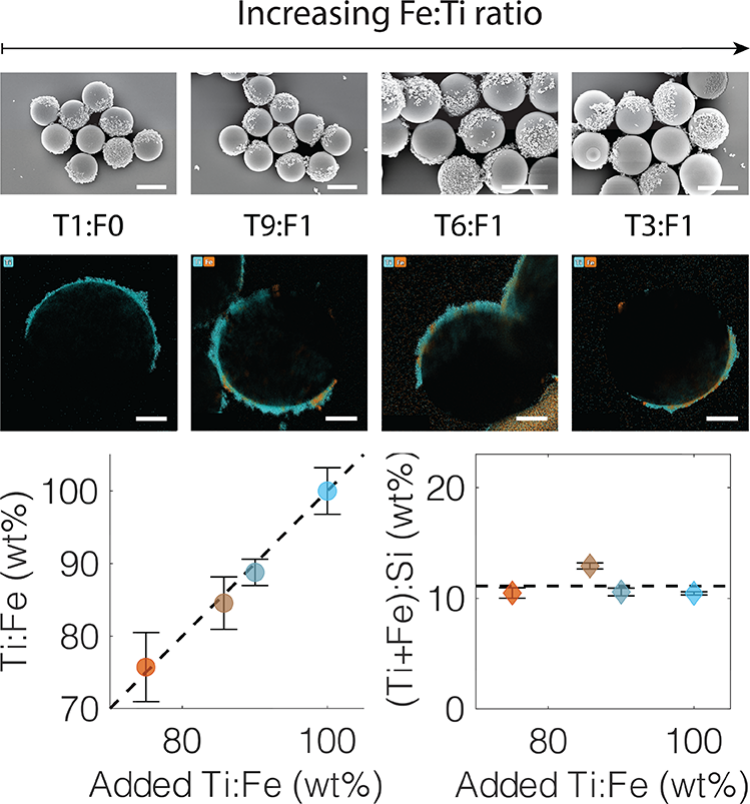
Synthesis of Janus microswimmers functionalized with TiO_2_ and Fe_2_O_3_ by TNA. Top and middle rows: an increasing amount of Fe_2_O_3_ relative to TiO_2_ P-25 is added during the nanoparticle-attachment step (left to right). TX:FY denotes the ratio of TiO_2_ (X) to Fe_2_O_3_ (Y). Middle row: STEM EDX elemental mapping visualizes the increasing presence of Fe_2_O_3_ with addition. Bottom row: Quantification of Ti and Fe loading with ICP-OES of the visualized particles. The detected Ti:Fe amounts are in agreement with the expected values, while the overall loading remains essentially constant, demonstrating control over the functionalization of the Janus microswimmers. Error bars correspond to the respective standard deviations of the values accounting for error propagation from the measurements. Scale bars on the SEM images (top row) denote 2000 nm. Scale bars on the STEM images (middle row) denote 500 nm.

Janus microswimmers are arguably the simplest and thus the most popular system to study the dynamics of active matter.^56^ Their directed motion without external flows and ability to induce micromixing, with the potential to overcome diffusion limitations, holds promise for applications spanning water remediation,^57^ synthetic chemistry,^45^ and smart drug delivery.^58^ For such applications, not only the enhanced recovery of microswimmers using magnetic fields,^27,49,50^ but also the ability to steer their motion by the same means is highly desirable.^59−61^ We therefore demonstrate an example of TNA’s modularity by asymmetrically attaching mixed compositions of photocatalytic TiO_2_ and magnetic Fe_2_O_3_ nanoparticles in a single step (see Figure 4).

**Figure 5:**
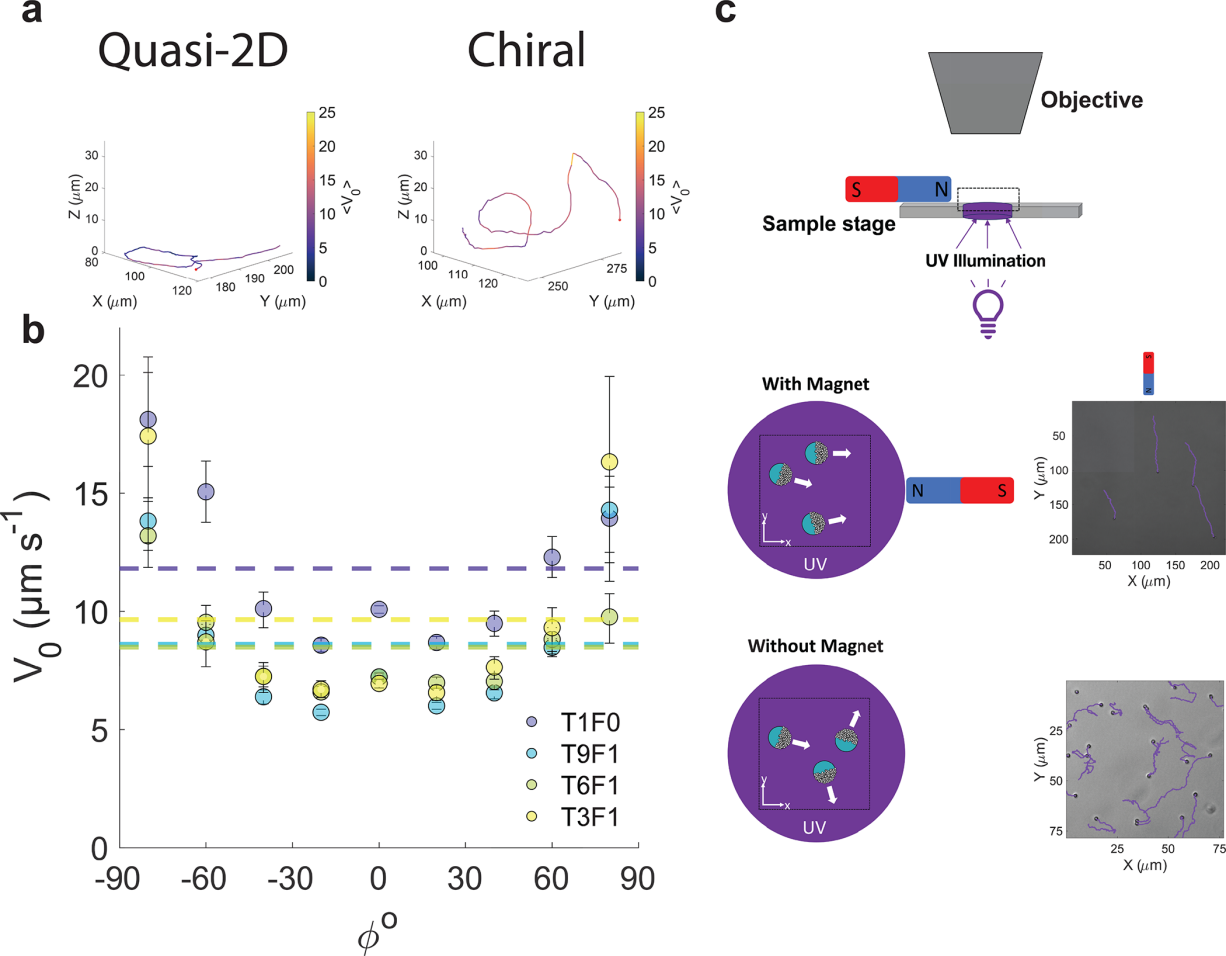
Motion of Fe_2_O_3_–TiO_2_–SiO_2_ obtained via TNA. (a) Examples of effective 2D and chiral 3D motion are highlighted, as tracked using ML. Color coding indicates the absolute value of the instantaneous velocity evaluated over 2 frames (0.2 s, in μms^–1^). Red dots indicate the starting point of the trajectory. (b) Instantaneous velocities of particles with different TiO_2_:Fe_2_O_3_, discretized by the angle made with the substrate between the direction of motion (see Figure S11 for further information). Confidence intervals are obtained by bootstrapping. Here, the data presented is from particles that are illuminated with UV (345 nm) in the absence of an applied magnetic field. (c) Decoupled steering and propulsion mechanisms of our Fe_2_O_3_–TiO_2_–SiO_2_ microswimmers (9:1, TiO_2_:Fe_2_O_3_). Top: schematic of the experimental setup. Bottom: trajectories of the catalytic Janus particles with and without magnet when illuminated with low intensity UV (365 nm). The particles swim in the direction of the magnetic field but exhibit randomly directed motion in its absence (see Figure S12 for further information on the experimental setup and control experiments).

As previously described, we find that Janus photoresponsive microswimmers obtained via TNA often display a “quasi-3D” motion under UV illumination, with extended “sliding-states”^62^ punctuated by rapid, out-of-plane ballistic segments of motion.^30^ We furthermore observe a range of swimming behaviors, ranging from a 2D motion typical of Janus catalytic microswimmers^42,62−65^ (see Figure 5a, “Quasi-2D”) to a highly chiral motion characterized by multiple loops in a “roller-coaster” like behavior (see Figure 5a, “Chiral”). To localize the particles in 3D and extract their instantaneous velocities, we use an in-house Machine Learning model (see Experimental Section, Figure S10, and ref (47) for more details), which we train on experimental data (26 924 total labeled observations). In agreement with previous experimental findings,^30^ we observe an orientation-dependent velocity with faster segments of motion out of plane in both directions compared to in-plane (see Figure 5b), contrasting with the results of ref (66) where particle self-shadowing plays a critical role in determining microswimmer motion. Here, the orientation of the particle motion with respect to the substrate is defined by the angle between the velocity vectors for in-plane and out-of-plane motion (see Figure S11 for further information). The underlying mechanisms contributing to this behavior will be the subject of future investigation; however, we expect a complex interplay between the shape and asymmetry of the microswimmers arising from the structure and coverage of the TiO_2_ nanoparticles,^67^ the rotational diffusion of the microswimmers arising from thermal fluctuations, the direction of illumination,^66^ and the sliding attractor states stemming from generated chemical gradients and hydrodynamic particle–substrate interactions.^62^ By separating the domains of magnetic and photocatalytic functionality, we do not expect any significant changes in swimming speeds, e.g., due to reduced charge recombination.^59^ We moreover emphasize that, for the trajectories reported in Figure 5a,b, no magnetic field is applied.

The magnetic responsiveness of our Janus microswimmers is demonstrated by a simple experiment. Under low UV illumination (to prevent 3D motion) and without the application of a magnetic field, the (T9F1) microswimmers translate in random directions based on the orientation of their photocatalytic cap (see Figure 5c, bottom). However, placement of a small magnet in combination with the low intensity UV light orients the microswimmers parallel to the applied magnetic field. This shows the ability of a small quantity of Fe_2_O_3_ magnetic nanoparticles to orient the microswimmer in a specified direction, in turn imparting control over their motion. Therefore, our modular approach to TNA enables the scalable fabrication of multifunctional Janus microswimmers with decoupled steering (magnetic) and propulsion (light) mechanisms, similar to the “colloidal-surfers” first demonstrated by Palacci et al.,^68^ and distinguishing itself from more typically studied systems where translational motion and steering are both controlled by the imposition of an externally applied magnetic field.^69,70^ Directing the orientation of the hybrid magnetic-catalytic caps could also be used to rectify their orientation and thus provide a further degree of control over microswimmer speed.^71^ We furthermore expect that the ability to modify the base photocatalysts with various cocatalyst nanoparticles previously outlined will open the door to a combinatorial range of new microswimmer architectures and behaviors, which nonetheless will demand future detailed mechanistic studies.^43^

## Conclusion

We have presented a versatile method to attach various presynthesized functional nanoparticles onto microparticle supports “off-the-shelf”. The flexibility of our strategy is inherent in the ability to encode chemical complexity into a polymer bridge, which allows the appropriate selection of functional groups to bind different metal and metal-oxide nanoparticles to target a desired application. In short, using the “Swiss-army-knife” pPFPAC polymer bridge enables a “pick’n’mix” approach to support nanoparticles onto various substrates, addressing some of the limitations currently facing the application of nanoparticles to, e.g., photocatalysis. Attaching plasmonic materials, e.g., Au nanoparticles, onto SiO_2_ microparticles could also find uses in imaging and sensing applications due to their favorable optical properties.^72^ Furthermore, the simplicity and gentle synthetic conditions of this strategy allows its application to Pickering wax emulsions, in turn enabling the modular synthesis of large quantities of multifunctional microswimmers via Toposelective Nanoparticle Attachment. Therefore, we hope our method will help bridge the fields of catalysis, nanochemistry, and active matter and thereby facilitate an interdisciplinary approach to the synthesis of designer microswimmers and thus bring the promise of “chemistry-on-the-fly”^44^ one step closer to realization.

## Experimental Section

### Materials

Common solvents and reagents were purchased from commercial suppliers (VWR, Acros, Sigma-Aldrich, Fluka, Fluorochem) at ≥95% quality and used as received. Extra dry tetrahydrofuran (THF) and dimethylformamide (DMF) (over molecular sieve) were used as supplied. Triethylamine (TEA) (VWR, technical purity) was distilled under N_2_ from KOH before use. The water used in all syntheses and in particle tracking experiments was taken from a Thermo Fisher GenPure Pro instrument with a resistivity ≥18MΩ·cm.

The synthesis of nitrodopamine and the precursor polymer poly(pentafluorophenyl acrylate) (pPFPAc) is detailed in our earlier publication.^30^

### Synthesis of the Polymer Bridge and Its Postmodification

Three variants of the polymer bridge (“macromolecular glue”) were synthesized for this work. For the first, a poly(hexane-6-amine, propyl-3-dimethyloxysilane, nitrodopamine)-acrylamide, which is tailored for the linkage of SiO_2_ surfaces to transition metal-oxides (titania and iron in our case here), the synthetic details can be found in our earlier work.^30^ The following two polymers were synthesized to bridge noble metals and mixtures of noble metals and transition metal-oxides:

#### Poly(hexyl-6-amine, Ethylthiol, Nitrodopamine)-Acrylamide

Pentafluorophenyl acrylate (pPFPAc) (502 mg, 2.1 mmol of active ester units) was dissolved in 10 mL of dry DMF under N_2_ at 50 °C. *N*-boc-Hexanediamine hydrochloride (265 mg, 1.05 mmol) was dissolved in 2 mL of dry DMF and subsequently added dropwise to the pPFPAc solution with an excess of triethylamine (0.45 mL, 3.23 mmol). After 1 h of stirring at 50 °C under N_2_, 1 mL of a solution of aminoethanethiol (40.5 mg, 0.52 mmol) was added dropwise followed with 0.2 mL of TEA. Nitrodopamine was added in excess after another reaction time of 1.5 h, i.e., 309 mg (1.05 mmol) in 2 mL of dry DMF combined with another 0.2 mL of TEA. The reaction vessel was left stirring overnight at 50 °C. DMF was reduced in a rotary evaporator, and the product was redissolved in 12 mL of DCM. The N-Boc group of this crude product was removed by adding 4 mL of TFA and stirring it overnight at room temperature. The solvents were evaporated; the polymer was dissolved in 20 mL of water and dialyzed against water (Spectrapor3 membrane, MWCO 3.5 kDa) over the course of a week with multiple water changes. To obtain the final yellow-brownish polymer, the final solution was freeze-dried. Yield: 278.9 mg.

The resultant polymer should therefore have a modification degree of 2:1:1 of amine, thiol, and nitrodopamine, respectively. This was checked by conversion measurements via ^19^F-NMR spectra (see Figure S13 and Table S3 for further information). Although ATR-FTIR measurements of the resultant polymer were inconclusive, postsynthetic analysis performed by ^1^H NMR indicates the presence of nitrodopamine (see Figure S14). To demonstrate the incorporation of the thiol group, a UV–vis analysis with Ellman’s reagent was performed as described in Figure S15.

#### Poly(hexyl-6-amine, Ethylthiol)-Acrylamide

The synthesis of the polymer bridge exclusively toward SiO_2_ and gold followed the same procedure as above, but with a target modification degree of 3:1 of hexylamine and ethylthiol, respectively.

### Decoration of TiO_2_ P-25 Nanoparticles with Au and Pt Nanoparticles

#### Decoration of TiO_2_ P-25 Nanoparticles with Au Nanoparticles via Deposition-Precipitation

TiO_2_ P-25 nanoparticles were modified with Au nanoparticles following the synthesis of Gualteros et al.^54^ Briefly, an aqueous suspension of TiO_2_ P-25 (Sigma-Aldrich, 21 nm primary size, 1 w/v%), urea (Sigma-Aldrich, 0.7w/v%), and HAuCl_4_·3H_2_O (Sigma-Aldrich, 0.5 w/v%) was heated to 90 °C and stirred at this temperature for 4 h. The solids were then collected and washed with water (4×) by centrifugation, before drying at 50 °C overnight under vacuum. Finally, the dried material was calcined at 300 °C with a ramp rate of 10 °C/min for 4 h, with a final purple color.

#### Decoration of TiO_2_ P-25 Nanoparticles with Pt Nanoparticles

TiO_2_ P-25 nanoparticles were decorated with presynthesized Pt nanoparticles by solvent evaporation.^15^ Briefly, a MeOH suspension of TiO_2_ P-25 (Sigma-Aldrich, 21 nm primary size, 0.2 w/v%) and Pt nanoparticles (Sigma-Aldrich, 3 nm, 0.01 w/v%) was prepared. The solvent was then slowly removed in a rotary evaporator at 50 °C, as the pressure was gradually reduced to a final pressure of 120 mbar. The resultant gray precipitate was then collected and dried for 2 h at 120 °C under vacuum.

### Supporting Functional Nanoparticles on SiO_2_ Microparticles via a Polymer Bridge

Polymer solutions were prepared by dispersing the dry poly(acrylamide)-based functional polymers (poly(acrylamide)-(1,6-hexanediamine, nitrodopamine), poly(acrylamide)-(1,6-hex-anediamine, 2-aminoethanethiol), or poly(acrylamide)-(hexane-6-amine, propyl-3-dimethyl-oxysilane, nitrodopamine), depending on the desired nanoparticles to be attached) in water at 50 °C overnight under stirring (100 mg/L). Cleaned SiO_2_ microparticles were prepared by adding 1 w/v% SiO_2_ aqueous suspensions to a bubbling 70 °C H_2_O_2_/NH_4_OH solution (1:1:1 volumetric ratio) under magnetic stirring for 10 min to activate hydroxyl groups on the SiO_2_ surface. The activated SiO_2_ particles were washed repeatedly with water by centrifugation and redispersion and then added dropwise under magnetic stirring to the prepared polymer solutions and left stirring overnight (final SiO_2_ concentration 0.1 w/v%). The polymer-modified SiO_2_ particles were then washed by centrifugation multiple times to remove excess polymer and redispersed in phosphate-buffered saline (PBS, pH 7.0).

Functional nanoparticles (TiO_2_ P-25 (Sigma-Aldrich, 21 nm primary size), Fe_2_O_3_ (Sigma-Aldrich, <50 nm primary size), Au (nanoComposix, 70 nm)) were then all firmly attached to the SiO_2_ microparticle supports in the same manner, by adding the nanoparticles to the polymer–SiO_2_ suspensions under magnetic stirring (600 rpm), and left mixing overnight (SiO_2_:metal-oxide nanoparticles 5:1 by mass, SiO_2_:Au nanoparticles 10:1 by mass).

In the case of the Fe_2_O_3_–TiO_2_ mixtures, the ratio of metal-oxide nanoparticles was adjusted to the desired value before addition to the SiO_2_ microparticle suspension. Finally, the nanoparticle functionalized SiO_2_ microparticles were washed extensively with alternating sonication and centrifugation steps to remove any excess nanoparticles not firmly attached to the SiO_2_ microparticles.

### Synthesis of Janus Microswimmers by Toposelective Nanoparticle Attachment

Janus microswimmers were synthesized via Toposelective Nanoparticle Attachment (TNA) following the protocol outlined in ref (30). Briefly, SiO_2_-wax Pickering emulsions were prepared by following the recipe of Perro et al.^29^ Paraffin wax (455 mg, M.P. 58–62 °C) was added to a 5 mL water suspension (5 w/v% cleaned SiO_2_ particles, 10.8 mg/L didodecyldimethylammonium bromide (DDAB)^73^), heated to 75 °C, and then stirred for 15 min at 3000 rpm before vigorous mixing at 11 000 rpm for 160 s using an IKA T-25 Digital Ultraturrax.^74^ After the emulsification step, the Pickering emulsion was immediately placed in an ice bath to rapidly solidify the particle-stabilized wax droplets. The emulsion was then washed by sedimentation and decanting in a 0.1 M NaCl solution to remove the cationic surfactant, before further washing in deionized water to remove dissolved salt ions. The SiO_2_-wax colloidosomes were dispersed overnight by gentle agitation in an aqueous solution of the poly(acrylamide)-(hexane-6-amine, propyl-3-dimethyloxysilane, nitrodopamine)-based polymer using an orbital mixer. The polymer-modified colloidosomes were then washed thoroughly in deionized water before redispersion in a phosphate-buffered saline (PBS, pH 7.0) suspension containing the functional metal-oxide nanoparticles (TiO_2_ P-25 (Sigma-Aldrich, 21 nm primary size), Fe_2_O_3_ (Sigma-Aldrich, <50 nm primary size)). After gentle mixing overnight, the nanoparticle-functionalized colloidosomes were collected by filtration and the wax was finally removed with chloroform to obtain the asymmetrically modified Janus microswimmers.

### 3D Tracking of Microswimmers with Extremely Randomized Decision Trees (ERTs)

The 3D motion of our microswimmers is tracked using an Extremely Randomized Decision Tree (ERT) ensemble model, as outlined in further detail in ref (47). Briefly, labeled data to train the model was first obtained from 280 μL of dilute aqueous suspensions of the TiO_2_–Fe_2_O_3_–SiO_2_ Janus particles in a flow-through cell (cell 137-QS; Hellma Analytics) with a light path length of 1 mm. Z-stacks of the sedimented Janus particles were taken, and the Z-slices serve as the labels for the image data. Particles were imaged on an inverted microscope (Nikon Eclipse Ti2e) under Köhler illumination with white light using a 40× objective (CFI S Plan Fluor ELWD 40XC) with adjustable collar (set to 1 mm), and Z-stacks were taken with an exposure time of 30 ms using a Hamamatsu C14440-20UP digital camera. To simulate the conditions of swimming experiments, the particles were also illuminated with UV (340 nm, without fuel), using a Lumencor SPECTRA X light engine as the excitation source through the objective (epifluorescence).

Particles on each Z-slice were localized using the *MATLAB* implementation of the Hough circle transform, and masks centered on the particles, whose dimensions are dependent on the particle size and imaging resolution, were obtained. From these masks, key image features (the radial pixel intensity profile from the particle center, the mask’s moment of inertia, and information on the horizontal profile of the mask) were extracted. Exploratory factor analysis was then performed using the python *factor_analyzer* package to identify underlying latent numerical features and reduce the degree of collinearity in the feature space. The labeled features extracted were then used as inputs for the model training with a randomly shuffled train-test hold-out of 80:20 (26 924 total observations, of which 21 539 were used for model training). The ERT was trained on the freely available *Scikit-learn* package,^75^ using the *Scikit-learn ExtraTreesRegressor* class.

A normalized model error of 0.019 (ϵ = *s*(*z*_measured_ – *z*_label_)/*Z*_total_ (where ϵ is the normalized error, *s*(*z*_measured_ – *z*_label_) is the sample standard deviation of the residuals, and *Z*_total_ is the total valid range of tracking, from ref (76))) was achieved on the test data set (5385 observations). The trained model was then applied to data extracted from particle tracking experiments performed in the presence of fuel, as described below.

### 3D Motion Experiments

280 μL solutions of the Fe_2_O_3_–TiO_2_–SiO_2_ in fuel-rich aqueous conditions (H_2_O_2_, 3% v/v, diluted from a stock 30% v/v solution (Acros Organics)) were prepared in a microscopy cell as previously described, and videos were taken at 10 FPS. To activate the TiO_2_ photocatalyst and induce swimming, particles were illuminated with UV (340 nm), using a Lumencor SPECTRA X light engine as the excitation source through the objective (epifluorescence). Particles were imaged 20 μm below their focal plane to maximize the effective range over which their 3D motion could be tracked. The *XY* positions of the particles were localized using the *MATLAB* implementation of the Hough circle transform, while the vertical positions were localized as described above using ML. Spurious trajectories, defined as those with a standard deviation of displacements in Z noisier than that expected by Brownian diffusion, were filtered out before analysis of the population dynamics.

## Supporting Information Available

Details of the magnetic-UV experimental setup used, control experiments performed, and further characterization of the synthesized materials (PDF)
