# Supplementary material for: Modular Attachment of Nanoparticles on Microparticle Supports via Multifunctional Polymers
Source: Chem Mater. 2023 Apr 26;35(9):3731–41. doi: 10.1021/acs.chemmater.3c00555 (PMC10173378; doi:10.1021/acs.chemmater.3c00555)
Supplement: Supplementary file 1 — cm3c00555_si_001.pdf [file cm3c00555_si_001.pdf]

Supporting Information: Modular attachment of nanoparticles on microparticle supports via multifunctional polymers

Maximilian R. Bailey,* Tobias A. Gmür, Fabio Grillo, and Lucio Isa*

Laboratory for Soft Materials and Interfaces, Department of Materials, ETH Zürich, Zürich 8093, Switzerland

E-mail: maximilian.bailey@mat.ethz.ch; lucio.isa@mat.ethz.ch

Phone: +41 44 633 63 76

The Supporting Information includes:

Fig. S1: XPS spectra of SiO_2-TiO_2-Fe_2O_3

Fig. S2: Quantitative estimation of the SiO_2-TiO_2-Fe_2O_3 system by XPS

Figure S3: Magnetic responsiveness of SiO_2 supports with Fe_2O_3 nanoparticles

Figure S4: Magnetic hysteresis curves of free and supported Fe_2O_3 nanoparticles

Figure S5: Photocatalytic activity of free and supported TiO_2 P-25 nanoparticles

Figure S6: Elemental analysis of SiO_2-TiO_2-Pt

Figure S7: Elemental analysis of SiO_2-TiO_2-Au

Figure S8: Nanoparticle (TiO_2 and Au) attachment controls with chemical functional groups

Figure S9: XPS spectra of SiO_2-TiO_2-Au

Figure S10: 3D tracking of microswimmers with machine learning

Figure S11: Definition of the swimming orientation of a microswimmer

Figure S12: Overview of experimental set-up used for UV + Magnetic field experiments

Figure S13: ^{19}F-NMR spectra of the post-polymerization modification steps

Figure S14: ^{1}H-NMR spectrum, post-synthesis

Figure S15: Identification of thiol groups using Ellman's reagent

Table S1: Quantitative estimation of the SiO_2-TiO_2-Fe_2O_3 system

Table S2: Quantitative estimation of the SiO_2-TiO_2-Au system

Table S3: ^{19}F-NMR conversion measurements

The elemental composition of the sample surfaces were analyzed by X-ray photoelectron spectroscopy (PHI Quantera SXM). In all cases, an Al target X-ray source with a monochromator (Al Ka, hν = 1486.7 eV) was used with a neutralizer and the pass energy was 140 ev for the survey scans and 55 eV for the detail scans. The XPS spectra were analyzed with CasaXPS (v2.3.16), but no curve fitting was succesful. All binding energies are uncorrected as measured and shifted due to the use of a neutralizer emission of 7 mA.

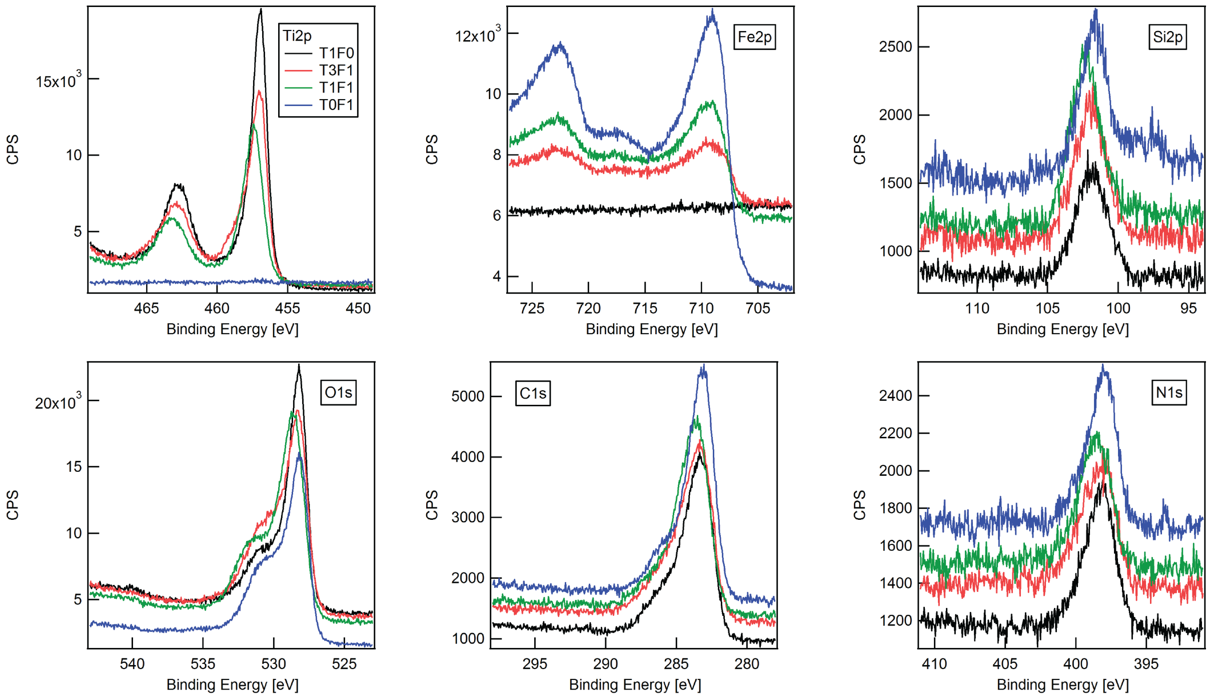

Figure. S 1: XPS detail spectra of the investigation of simultaneous adsorption of TiO_2- and Fe_2O_3-nanoparticles onto SiO_2 microparticles. Four ratios of Ti:Fe were investigated (1:0, 3:1, 1:1, and 0:1).

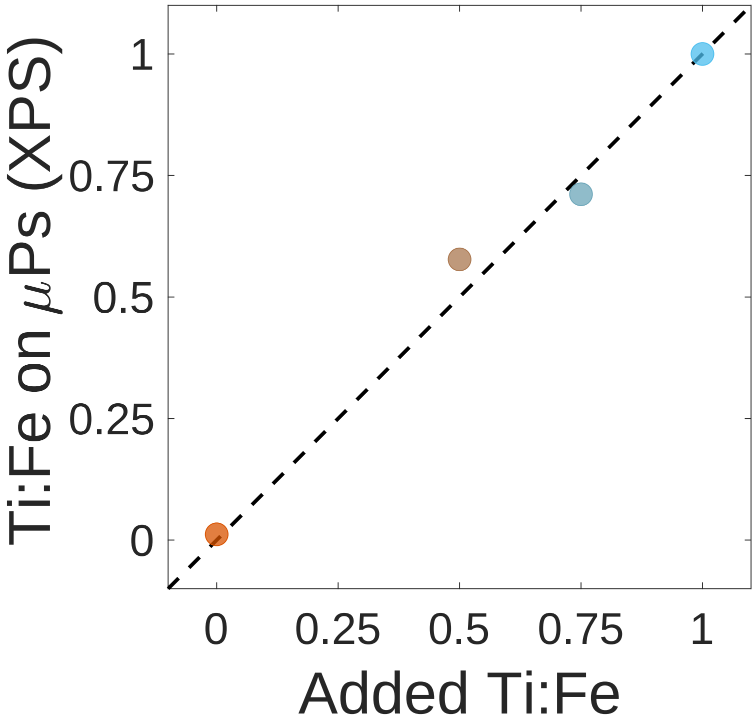

Figure. S 2: Scatter plots comparing the surface atomic ratio of Ti:Fe detected by XPS (tabulated data in Table S1) to the added Ti:Fe mass ratio. Given the respective molecular weights of TiO_2 and Fe_2O_3 per mole of Ti and Fe, we note that these values can be directly compared.

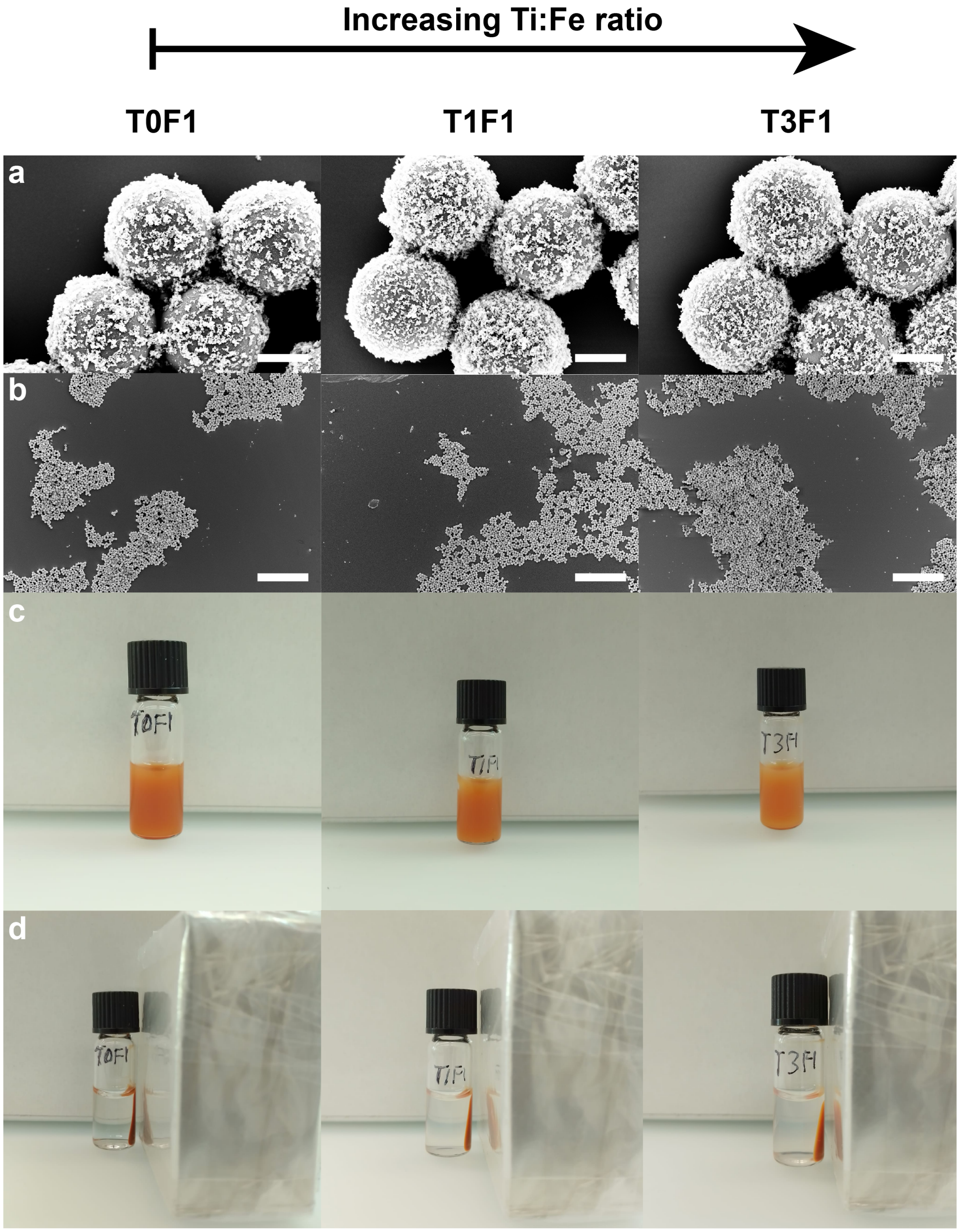

Figure. S 3: Magnetic responsiveness of SiO_2 microparticle supports with Fe_2O_3 nanoparticles attached in different ratios to TiO_2 P-25 nanoparticles. In all cases, we note that the Fe_2O_3 nanoparticles retain their magnetic responsiveness after attachment to the SiO_2 supports. a) Close up view of the functional nanoparticles supported on the microparticle supports. Scale bars represent 1 μm b) Zoomed out overview of the particles, demonstrating absence of free nanoparticles in solution. Scale bars represent 50 μm c) Dispersed microparticles in absence of magnetic field. d) Responsiveness of microparticle supports to an applied magnetic field. The particles rapidly collect towards the source of the magnetic field.

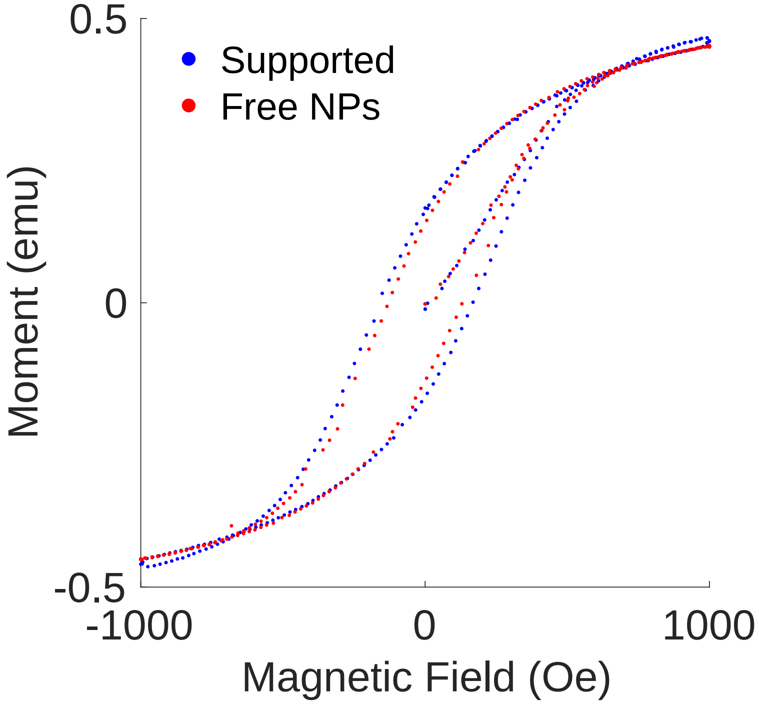

Figure. S 4: Magnetic hysteresis curves of the free (red circles) and microparticle supported (blue circles) Fe_2O_3 nanoparticles are obtained using a Vibrating Sample Magnetometer (VSM) at 300K. The magnetic moment of the Fe_2O_3 nanoparticles supported on the SiO_2 microparticles have been rescaled to be comparable to the equivalent mass of free nanoparticles (determined by ICP-OES). We observe good agreement between the 2 hysteresis curves, indicating that the magnetic properties of the nanoparticles are not affected by the attachment process. Slight variations can be accounted by weighing error of the samples and slight losses of material during transfer into the sample holder.

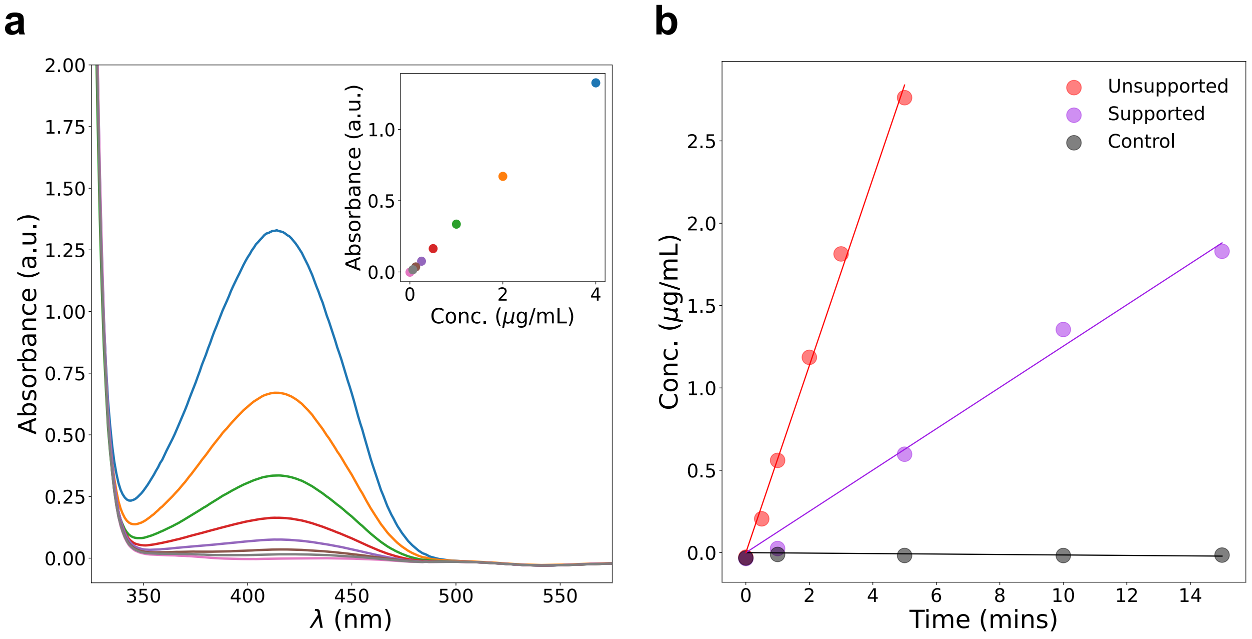

Figure. S 5: We quantify the photocatalytic activity of the free and supported TiO_2 P-25 nanoparticles by evaluating the 0th order methanol oxidation reaction, as described in[1] . Specifically, we use the Nash method[2] to estimate the formation of Formaldehyde, applying the Beer-Lambert law (see Figure 5 a). We find that the TiO_2 nanoparticles supported on the SiO_2 microparticles produce over 4x less formaldehyde than an equivalent mass of the free, unsupported nanoparticles (see Figure 5 b). We note the turbidity of the supported microparticle suspension, and suppose that the reduced transmission of UV light is one of several factors contributing to the reduced activity, as well as the reduced exposed surface area of the supported nanoparticles due to their contact points with the SiO_2 microparticles. Nevertheless, the supported TiO_2 nanoparticles retain their photocatalytic properties, as also demonstrated by their motility under UV light in the presence of H_2O_2.

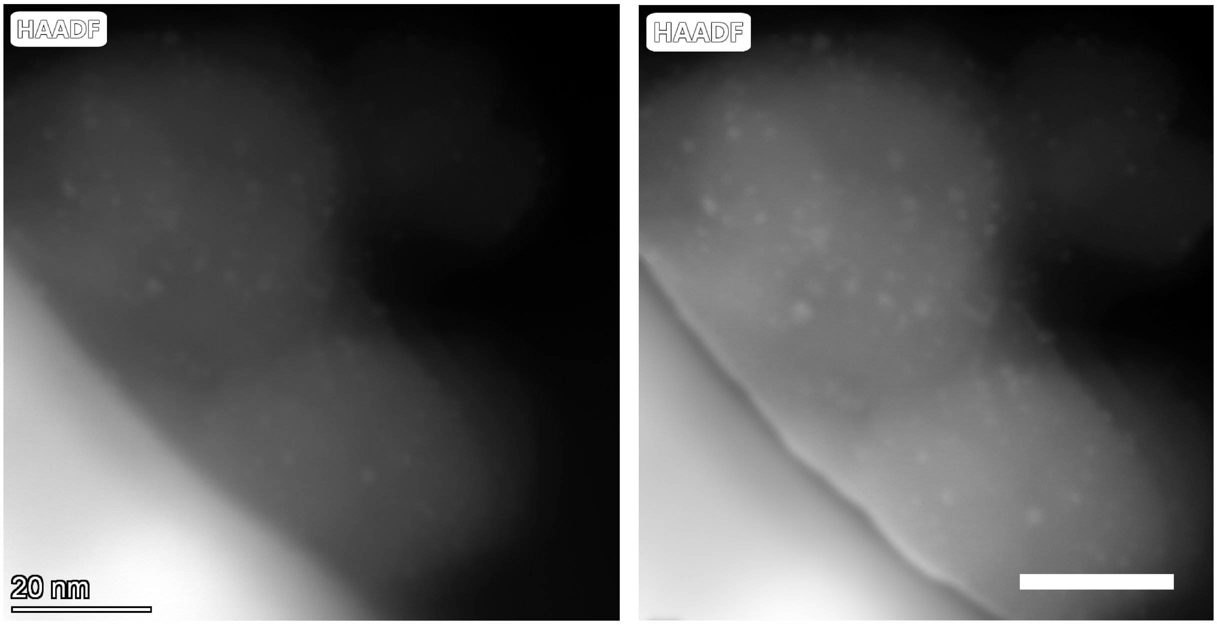

Z	Element	Family	Net intensity	Net background	K-factor	Absorption correction	Atomic fraction	Mass fraction	Fit error
-	-	-	counts	counts	-	-	%	%	%
8	O	K	7.55627E+05	9.83991E+03	1E+00	1E+00	5.66166E+01	3.44668E+01	1.97971E-01
14	Si	K	5.08313E+05	1.69494E+04	8.85161E-01	1E+00	1.92048E+01	2.05233E+01	2.53615E-01
22	Ti	K	9.31398E+05	2.973E+04	1.02911E+00	1E+00	2.4005E+01	4.37211E+01	1.20137E-01
78	Pt	L	1.29198E+04	4.74834E+04	2.05031E+00	1E+00	1.73627E-01	1.28878E+00	2.74771E-01

Figure. S 6: EDS of SiO_2-TiO_2-Pt, visualised using STEM-HAADF imaging. Top Left: Raw image data, as obtained. Top right: Contrast-enhanced image used in the main text. While it is not possible to directly map the presence of the Pt nanoparticles due to their small size, they appear as brighter spots in HAADF imaging, and the L-edge of Pt is detected from EDX (bottom row, tabulated values).

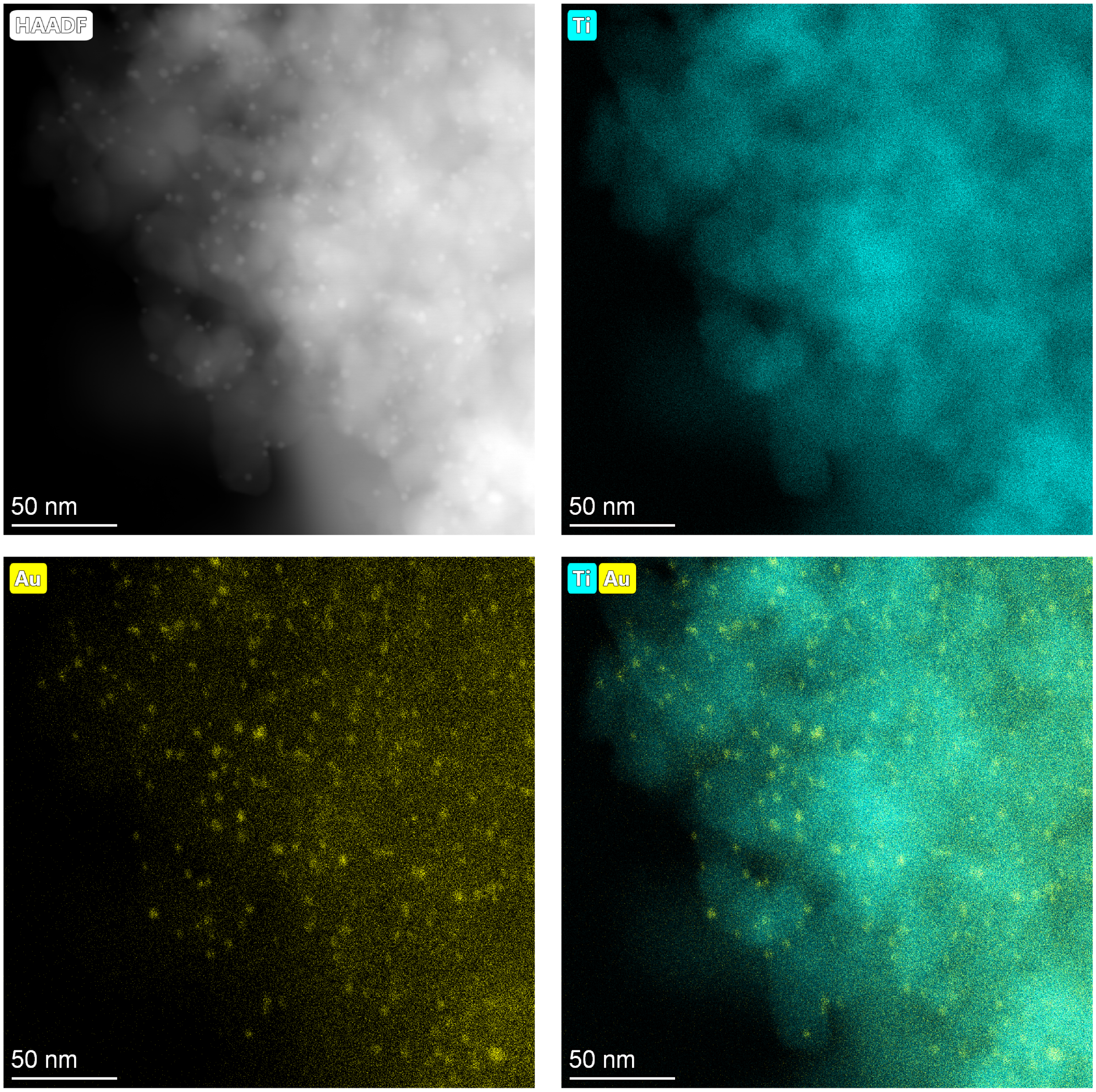

Z	Element	Family	Net intensity	Net background	K-factor	Absorption correction	Atomic fraction	Mass fraction	Fit error
-	-	-	counts	counts	-	-	%	%	%
8	O	K	3.68822E+06	1.94867E+04	1E+00	1E+00	5.84616E+01	3.90274E+01	1.00214E-01
14	Si	K	3.39259E+06	7.81563E+04	8.85161E-01	1E+00	2.71162E+01	3.17765E+01	7.02579E-02
22	Ti	K	2.6336E+06	8.30049E+04	1.02911E+00	1E+00	1.43593E+01	2.86791E+01	6.02901E-02
79	Au	L	2.17381E+04	8.89529E+04	2.10799E+00	1E+00	6.29045E-02	5.16975E-01	1.21035E-01

Figure. S 7: EDS mapping and HAADF-STEM imaging of SiO_2-TiO_2-Au. HAADF-STEM image, and the corresponding EDS mapping for Ti, Au, and Ti-Au overlayed are shown sequentially. The EDS mapping images shown represent the integrated values during acquisition, which is chosen for visualisation purposes. Elemental analysis for the image is presented in the bottom row (tabulated values).

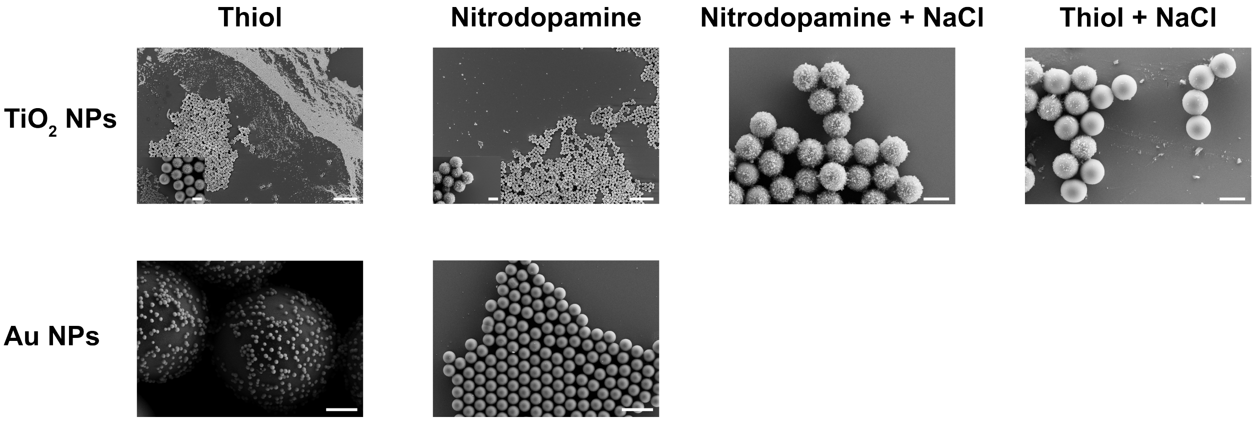

Figure. S 8: Controls for the attachment of nanoparticles (TiO_2, Au) via different chemical functional groups (nitrodopamine, thiol). The attachment of Au nanoparticles requires the presence of thiol groups, evidenced by the lack of Au nanoparticle attachment using only nitrodopamine (see bottom row). Some attachment of TiO_2 nanoparticles is observed in the absence of the nitrodopamine functionality. However, the nanoparticles are not firmly attached, as observed by the large scale removal of TiO_2 nanoparticles from the SiO_2 microparticle supports after drying in absence of the nitrodopamine functionality (compare top row, left, with top row, centre left - the white bands across the substrate in the thiol control are detached free nanoparticles). We expect that some TiO_2 attachment is achieved via electrostatic aggregation (through the quarternary ammonium group), however these nanoparticles are not bound well in the absence of the nitrodopamine group. We perform a further washing step in NaCl (0.1M), and find that the TiO_2 nanoparticles remain well attached only in the presence of nitrodopamine (compare top row, centre right, with top row, right). We therefore conclude that thiol is necessary for the attachment of Au nanoparticles, and nitrodopamine is necessary for the stable attachment of metal-oxide nanoparticles. Top row (left to right): Scale bars represent 20 μm (inset 2 μm), 20 μm (inset 2 μm), 2 μm, and 2 μm respectively. Bottom row (left to right): Scale bars represent 500 nm and 6 μm respectively.

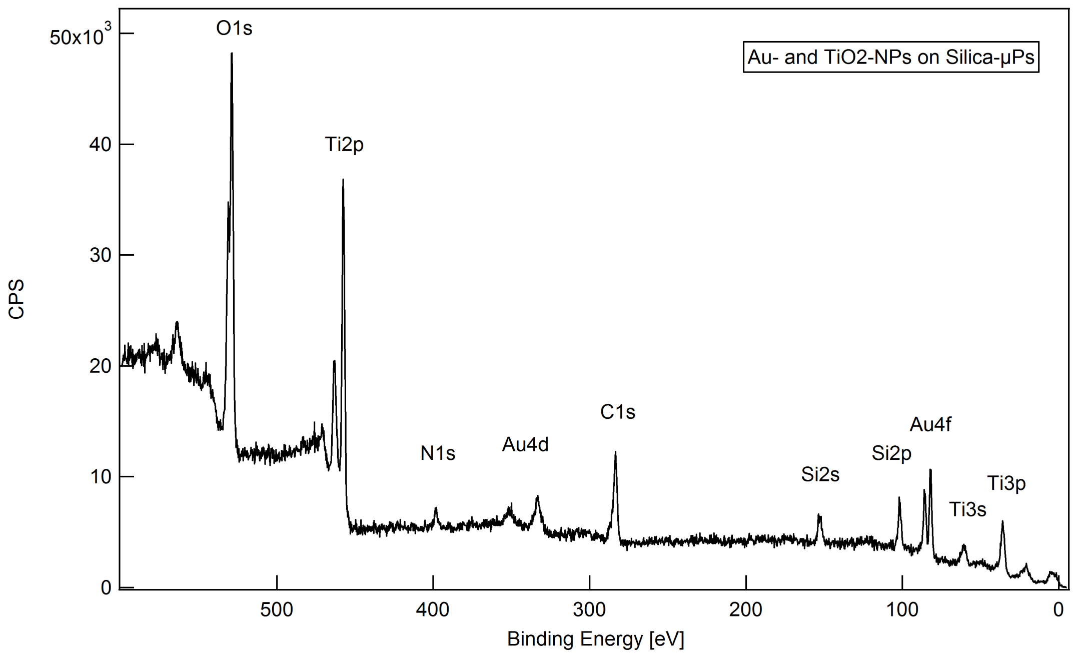

Figure. S 9: XPS survey spectra of the investigation of simultaneous adsorption of TiO_2- and Au-nanoparticles onto SiO_2 microparticles. The characteristic peaks of the expected elements are labelled and quantified below. No additional contamination is evident.

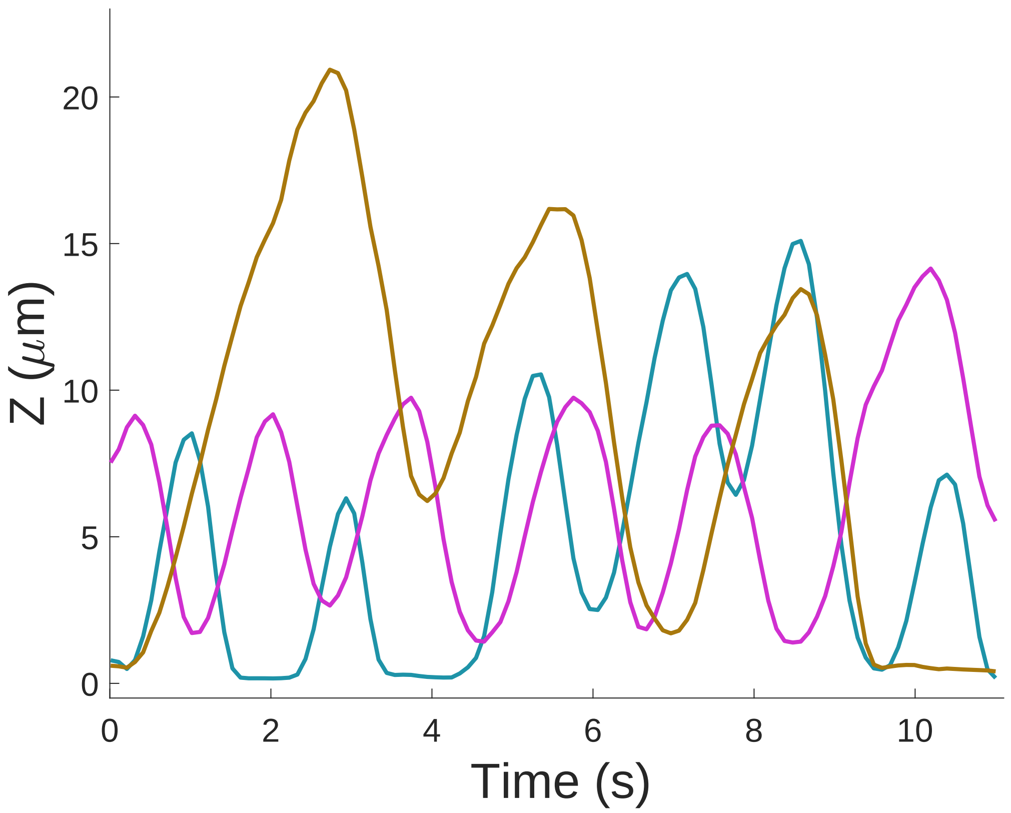

Figure. S 10: Tracking the 3D motion of Janus microswimmers using an Extremely Randomised Decision Tree (ERT) model. Selected trajectories showing Z positions vs time (different colours denote different microswimmers), comparing their out-of-plane motion. The trajectories are smoothed with a 3-point moving average for visualisation. For more information on the model and its training, see.[3]

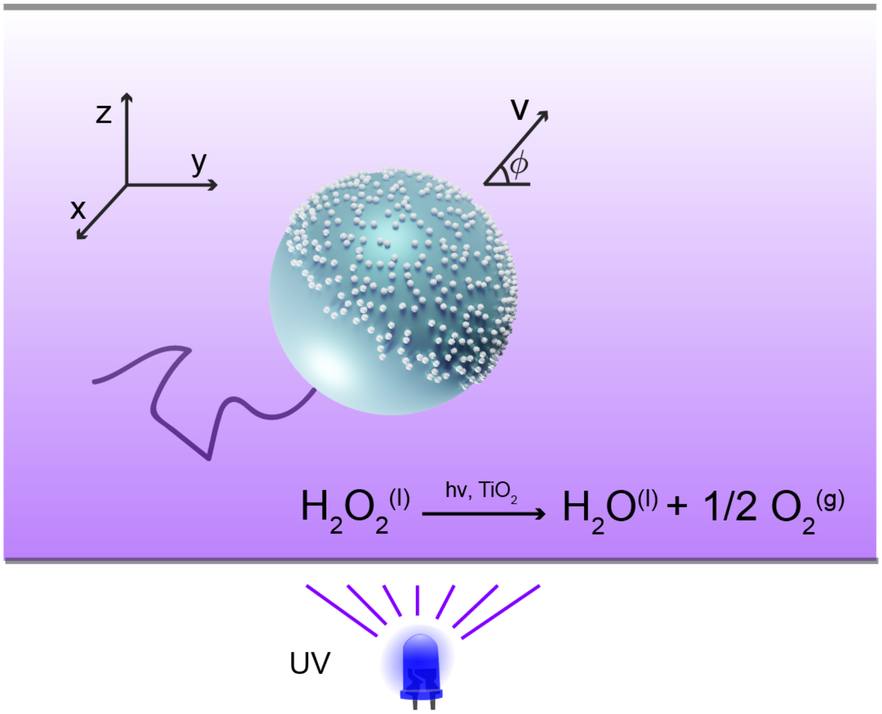

Figure. S 11: Definition of swimming direction used to discretise instantaneous velocities with orientation in Figure 5 of the main text (scheme reproduced from[4]). By decomposing hydrogen peroxide, the microswimmers swim with the functionalised cap forwards (or backwards, dependent on the solution pH[5])

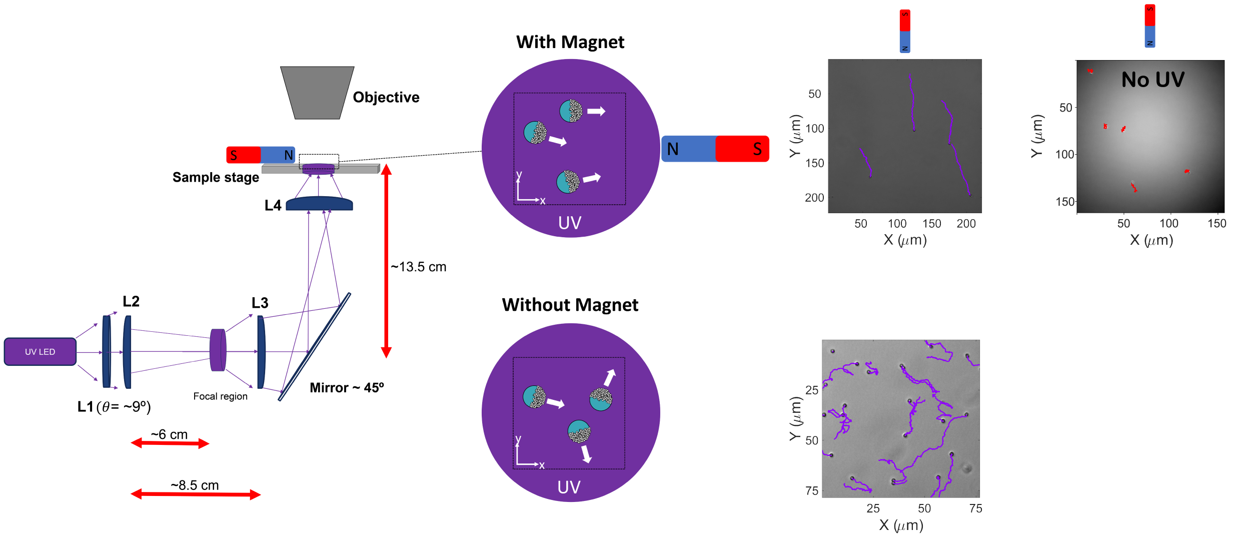

Figure. S 12: Overview of the experimental set up used to demonstrate control of the swimming direction of the microswimmers (propelled via UV illumination) using a magnetic field. L1 is an aspherical, collimating lens, while L2 and L3 are piano-convex lenses of the intermediate Kepler telescope. L4 is the final condenser lens to focus the UV source onto the sample. The design of the illumination system was inspired from.[6] The magnetic functionality of the Janus microswimmers enables alignment with the applied magnetic field, in turn imparting steering functionality as the motion of direction is dictated by the cap orientation of the microswimmers.[5] The characteristic, "persistent random walk"[7] of the microswimmers in the absence of the applied magnetic field is also shown for reference. Finally, we also demonstrate in the presence of the magnetic field, but in the absence of the UV light, the particles display Brownian diffusive motion, indicating that the magnetic field does not drive translation and the propulsion is thus decoupled from the steering mechanism.

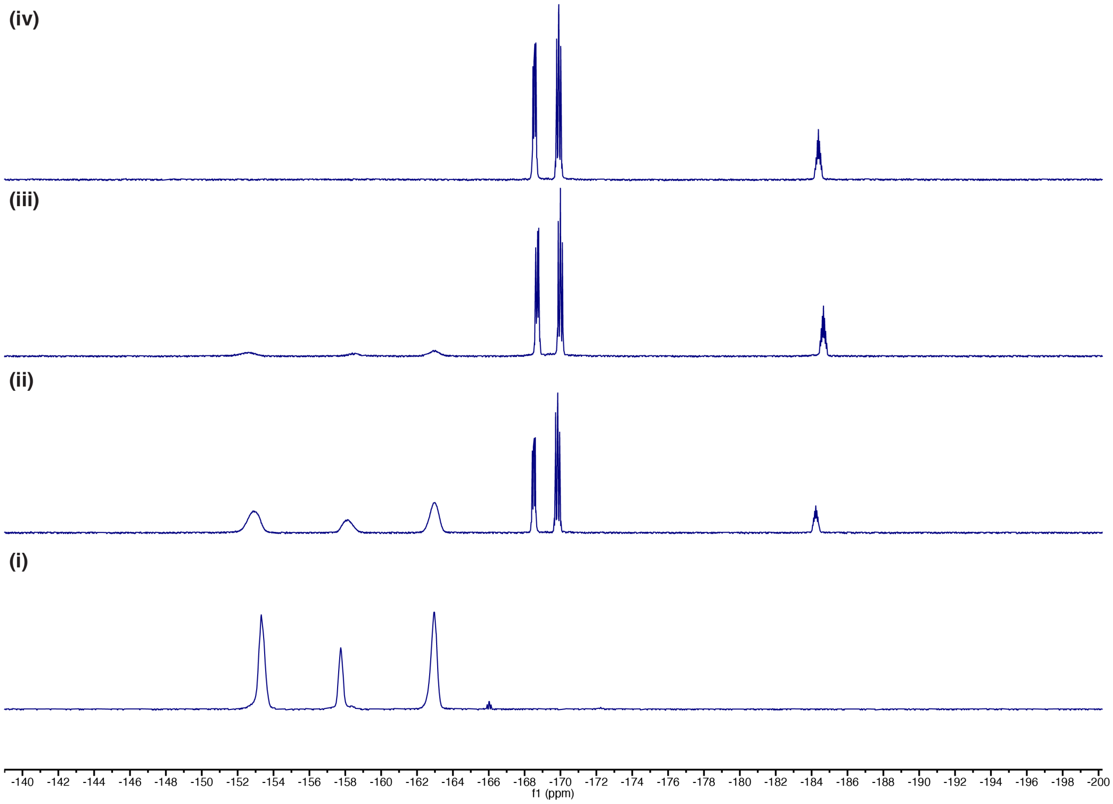

Figure. S 13: ^{19}F-NMR (188 MHz, $CDCl_3$) spectra of aliquots taken during the following post-polymerization modification steps: i) just before the addition of N-boc-hexanediamine hydrochloride ii) 1 hour after addition of N-boc-hexanediamine hydrochloride. iii) 1.5 hours after the addition of aminoethanethiol (iv) after the addition of nitrodopamine

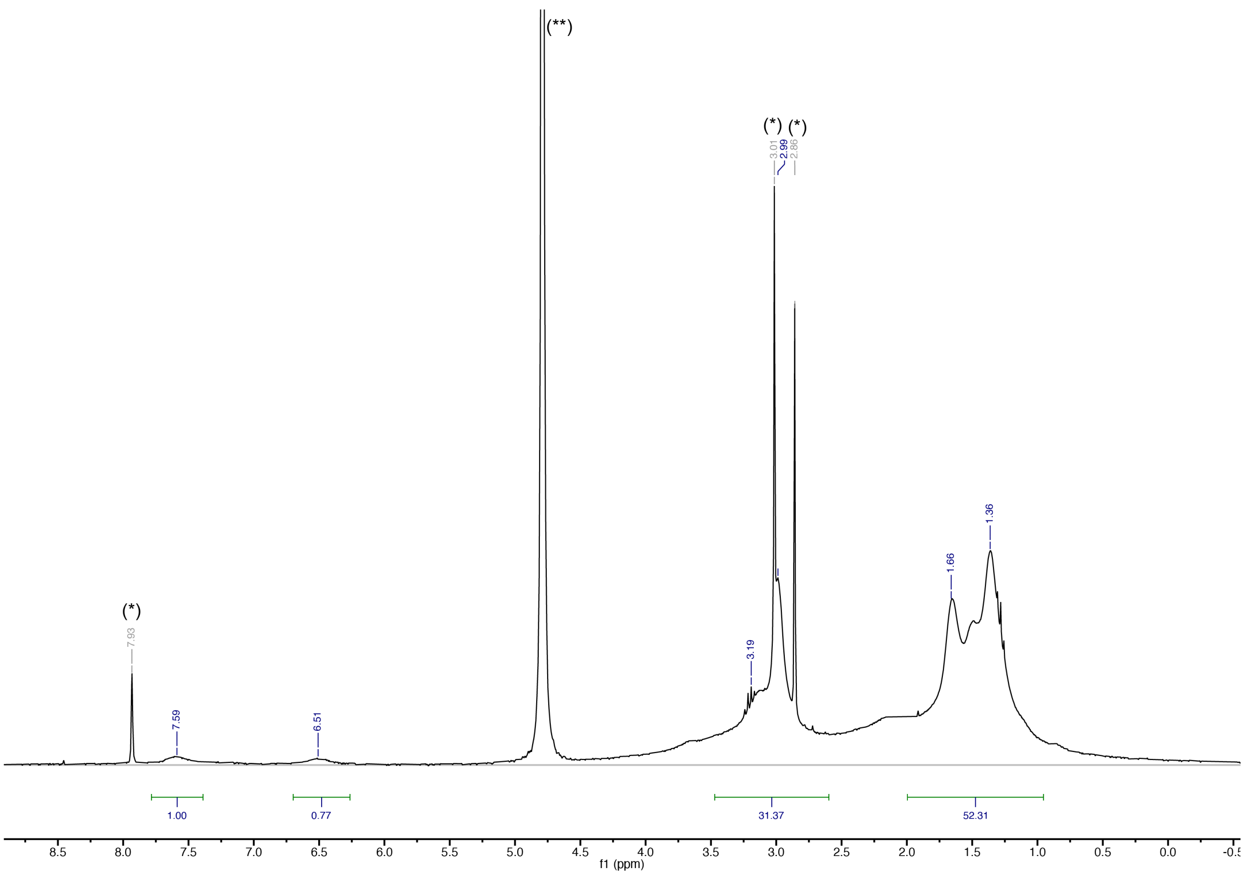

Figure. S 14: ^{1}H-NMR (300 MHz, D_2O) spectrum. (*) Residual DMF (**) H_2O. The peaks at 7.59 and 6.51 ppm are characteristic for nitrodopamine, the thiol characteristic peaks are not identifiable.

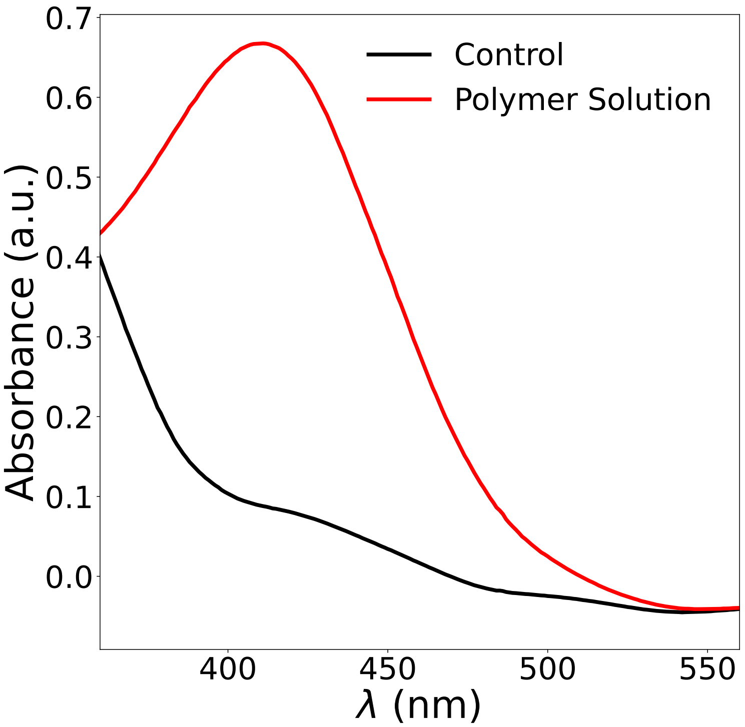

Figure. S 15: UV-Vis spectra of the thiolated polymer and a reference solution after reaction with Ellman's reagent.[8] The polymer required reactivation of its thiol groups after storage, necessitating the use of a reducing agent ($NaBH_4$). To minimise unwanted side reactions, the reaction with Ellman's agent and subsequent measurement is performed 4.5 hours after addition of the reducing agent. However, we still note that in the reference (containing only the equivalent amount of $NaBH_4$ in water), TNB^{2-} is produced continuously, albeit at a much slower rate (see relative ratio of peaks at 412 nm at the given measurement time). Due to these various sources of error, we therefore refrain from providing a quantitative value of the amount of thiol groups present in the polymer. Nevertheless, the much more rapid production of TNB^{2-} in the presence of the polymer is indicative of the presence of sulfur groups. It was not possible to perform the same measurement for the polymer containing both nitrodopamine and thiol groups, due to the overlap in the peak absorbance between nitrodopamine and TNB^{2-}.

Table S 1: Semi-quantitative estimation of the atomic concentration of the surface layer of SiO_2-TiO_2-Fe_2O_3 assemblies from the XPS spectra presented in Figure S1. Relative sensitivity factors correcting for the instruments' work function and pass energy are used as supplied by the instrument manufacturers.

	Ti1 : Fe0	Ti3 : Fe1	Ti1 : Fe1	Ti0 : Fe1
Ti 2p	14.1	10.6	9.7	0.2
Fe2p3	0.0	4.3	7.1	16.7
Si 2p	5.3	5.8	5.9	5.4
O 1s	44.7	50.9	52.2	43.9
C 1s	23.9	23.8	25.8	26.7
N 1s	3.4	3.4	2.8	3.7

Table S 2: Semiquantitative estimation of the surface layer of a SiO_2- TiO_2-Au assembly.

Peak:	C 1s	O 1s	Si 2p	N 1s	Ti 2p	Au 4f
at.%:	23.6	51.1	7.4	3.3	13.4	1.2

Table S 3: Quantification derived from integration of the ^{19}F-NMR measurements depicted in Figure S13. The results are to be taken with caution, since the background subtraction and phase correction were performed manually, and the reaction is also prone to hydrolysis.

step no.	nominal conversion(%)	measured conversion(%)
0	0	0
1	50	39.5
2	75	82.0
3	100	100

References

(1) La Zara, D.; Bailey, M. R.; Hagedoorn, P.-L.; Benz, D.; Quayle, M. J.; Folestad, S.; van Ommen, J. R. Sub-nanoscale surface engineering of Tio2 Nanoparticles by molecular layer deposition of poly(ethylene terephthalate) for suppressing photoactivity and enhancing dispersibility. *ACS Applied Nano Materials* **2020**, *3*, 6737–6748.

(2) Nash, T. The colorimetric estimation of formaldehyde by means of the Hantzsch reaction. *Biochemical Journal* **1953**, *55*, 416–421.

(3) Bailey, M. R.; Grillo, F.; Isa, L. Tracking Janus Microswimmers in 3D with machine learning. *Soft Matter* **2022**, *18*, 7291–7300.

(4) Bailey, M. R.; Grillo, F.; Spencer, N. D.; Isa, L. Microswimmers from Toposelective Nanoparticle Attachment. *Advanced Functional Materials* **2022**, *32*, 2109175.

(5) Singh, D. P.; Choudhury, U.; Fischer, P.; Mark, A. G. Non-Equilibrium Assembly of Light-Activated Colloidal Mixtures. *Advanced Materials* **2017**, *29*, 1701328.

(6) Vialetto, J.; Anyfantakis, M.; Rudiuk, S.; Morel, M.; Baigl, D. Photoswitchable dissipative two-dimensional colloidal crystals. *Angewandte Chemie International Edition* **2019**, *58*, 9145–9149.

(7) Löwen, H. Inertial effects of self-propelled particles: From active Brownian to active Langevin motion. *The Journal of Chemical Physics* **2020**, *152*, 040901.

(8) Riener, C. K.; Kada, G.; Gruber, H. J. Quick measurement of protein sulfhydryls with Ellman's reagent and with 4,4-dithiodipyridine. *Analytical and Bioanalytical Chemistry* **2002**, *373*, 266–276.
